# Paradigm Shifts in Perioperative Management of Colorectal Cancer: Personalization Based on Tumor Biology, Primary Site, and Recurrence Risk, and the Evolving Role of Organ Preservation

**DOI:** 10.3390/ijms27167261

**Published:** 2026-08-14

**Authors:** Kaoru Yoshikawa, Akira Ooki, Eiji Shinozaki, Eiichiro Toyokawa, Keito Suzuki, Manabu Shiozawa, Shin Maeda, Kensei Yamaguchi, Hiroki Osumi

**Affiliations:** 1 Department of Gastroenterological Chemotherapy, Cancer Institute Hospital, Japanese Foundation for Cancer Research, Tokyo 135-8550, Japan; kaoru.yoshikawa@jfcr.or.jp (K.Y.); akira.oki@jfcr.or.jp (A.O.); eiji.shinozaki@jfcr.or.jp (E.S.); eiichirou.toyokawa@jfcr.or.jp (E.T.); keito.suzuki@jfcr.or.jp (K.S.); kensei.yamaguchi@jfcr.or.jp (K.Y.); 2 Department of Colorectal Surgery, Kanagawa Cancer Center, Yokohama 241-8515, Japan; shiozawa.0m702@kanagawa-pho.jp; 3 Department of Gastroenterology, Yokohama City University Graduate School of Medicine, Yokohama 236-0004, Japan; smaeda@yokohama-cu.ac.jp; 4 Department of Gastroenterology, Kanagawa Cancer Center, Yokohama 241-8515, Japan

**Keywords:** perioperative therapy, total neoadjuvant therapy, watch-and-wait, mismatch repair deficiency, immune checkpoint inhibitor, organ preservation, circulating tumor DNA (ctDNA), minimal residual disease (MRD), colorectal cancer, rectal cancer

## Abstract

Perioperative treatment for colorectal cancer (CRC) is undergoing a paradigm shift from uniform cytotoxic regimens toward strategies guided by tumor location, microsatellite instability (MSI)/mismatch repair (MMR) status, and recurrence risk. Four clinically relevant subgroups now shape decision-making: microsatellite stable (MSS)/proficient MMR (pMMR) colon cancer, microsatellite instability-high (MSI-H)/deficient MMR (dMMR) colon cancer, MSS/pMMR rectal cancer, and MSI-H/dMMR rectal cancer. In MSS/pMMR colon cancer, adjuvant therapy is being refined through risk-adapted treatment duration and selective use of neoadjuvant chemotherapy. In MSS/pMMR rectal cancer, total neoadjuvant therapy, selective omission of pelvic radiotherapy, and watch-and-wait strategies are redefining treatment sequencing and organ preservation. In MSI-H/dMMR colon cancer, immune checkpoint inhibitor (ICI) therapy has shown marked activity in both adjuvant and neoadjuvant settings. In MSI-H/dMMR rectal cancer, neoadjuvant ICI therapy is being explored as a non-operative organ preservation strategy. In parallel, circulating tumor DNA (ctDNA)-based minimal residual disease (MRD) assessment is emerging as a tool for postoperative escalation, de-escalation, and surveillance. Across these settings, the maturity of the evidence varies widely, ranging from established phase III standards to guideline-endorsed but still single-arm approaches and investigational strategies that require prospective validation before routine adoption. This review summarizes the current evidence and remaining challenges in biology-, site-, and risk-adapted perioperative management of CRC.

## 1. Introduction

Colorectal cancer (CRC) is among the most commonly diagnosed malignancies worldwide. Approximately two million new cases were reported in 2020, and the International Agency for Research on Cancer (IARC) estimates that global incidence will increase to approximately 3.2 million cases by 2040, with deaths rising to approximately 1.6 million [1]. Over the past decade, advances in research and clinical care have been associated with improvements in overall survival [1]. At the same time, the rapidly expanding therapeutic landscape has made clinical decision-making increasingly complex, underscoring the need for continual, evidence-based updates.

In patients with resectable CRC, perioperative management has evolved in a stepwise manner, from surgery alone to postoperative adjuvant fluoropyrimidine-based therapy (5-fluorouracil/leucovorin), and subsequently to the establishment of oxaliplatin-based doublets (FOLFOX/CAPOX) as the standard of care [2,3]. Historically, perioperative treatment was guided by relatively uniform assumptions regarding treatment intensity and duration; however, these assumptions are now being reconsidered. Current paradigms increasingly emphasize individualized perioperative management based on tumor location (colon vs. rectum), recurrence risk, and tumor biology, particularly microsatellite instability (MSI)/mismatch repair (MMR) status. Importantly, individualized perioperative management is not synonymous with universal treatment intensification. Instead, treatment intensity and sequencing are increasingly tailored to tumor biology and recurrence risk. In lower-risk settings, this may support shorter adjuvant chemotherapy, whereas in selected patients with major or complete responses to preoperative therapy, non-operative management (NOM) with close surveillance may be considered.

In colon cancer, risk-adapted stratification has supported the consideration of shorter durations of adjuvant chemotherapy in selected lower-risk patients [4]. In rectal cancer, total neoadjuvant therapy (TNT) has redefined perioperative sequencing [5,6], and NOM has emerged as a relevant clinical option in appropriately selected patients [7]. In addition, response-adapted approaches that start with neoadjuvant chemotherapy and reserve chemoradiotherapy for inadequate responders have been reported as a means to explore radiotherapy omission in selected cases [8]. Furthermore, in microsatellite instability-high (MSI-H)/deficient mismatch repair (dMMR) colon cancer, neoadjuvant immunotherapy has demonstrated high pathological response rates [9,10], and preoperative immunotherapy activity has also been reported in MSI-H/dMMR rectal cancer [11].

**Figure 1:**
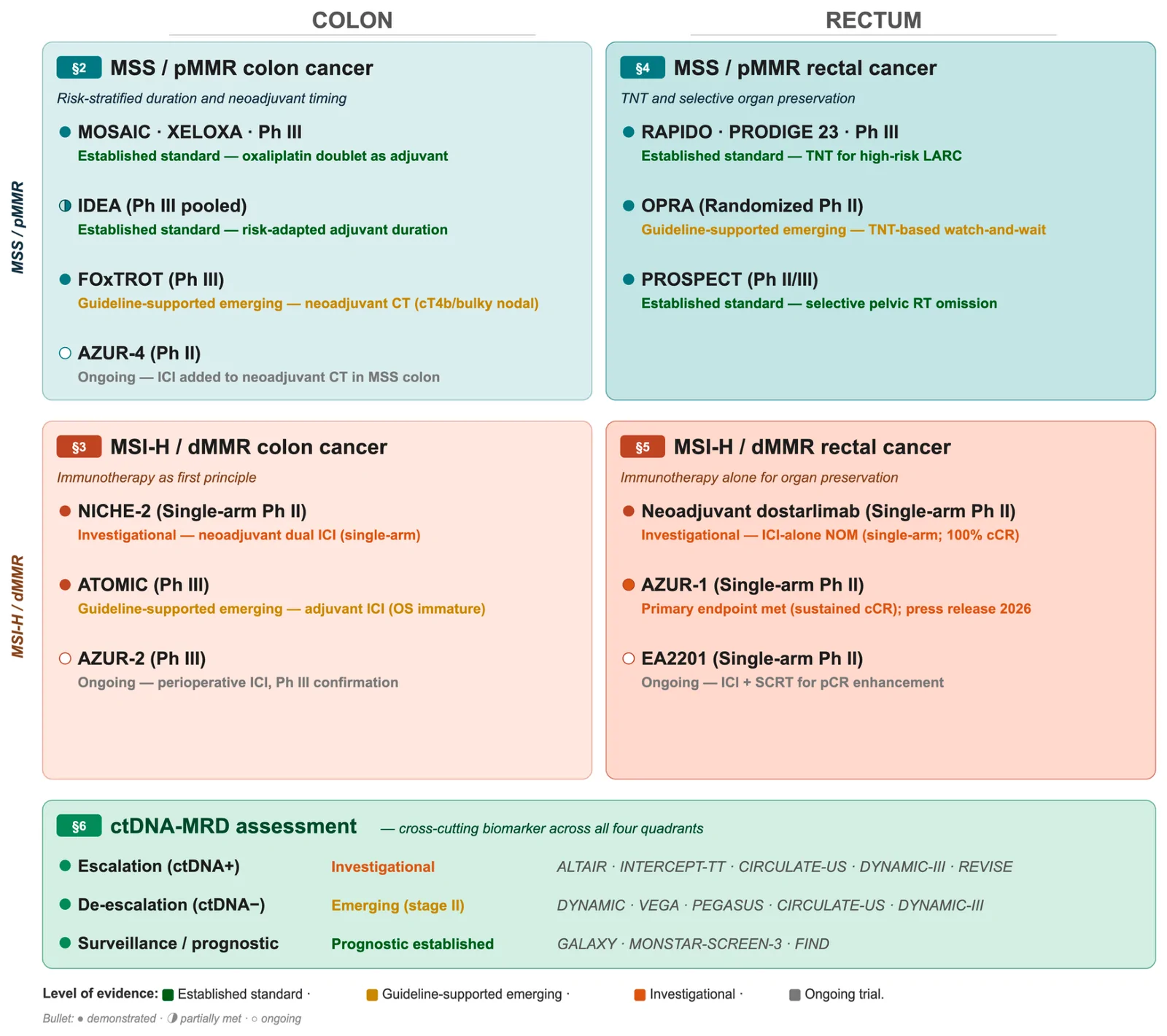
Four-quadrant framework for perioperative management of colorectal cancer, stratified by mismatch repair status and primary site. Trials shown: MOSAIC [2,12]; NO16968/XELOXA [3]; IDEA [4]; FOxTROT [13,14]; NICHE-1 [9]; NICHE-2 [10,15]; NICHE-3 [16]; ATOMIC [17]; RESET-C [18]; AZUR-4 [19]; PRODIGE 23 [6,20]; RAPIDO [5,21]; OPRA [7]; PROSPECT [8]; cohort data for MSI-H/dMMR rectal cancer [11,22]; AZUR-2 [23].

In this review, we conceptualize perioperative chemotherapy, radiotherapy, and immunotherapy within a unified multimodality framework and synthesize the available evidence. Our analytic framework is defined by MSI/MMR status and tumor location, and the discussion is structured around four categories: microsatellite stable (MSS)/proficient mismatch repair (pMMR) colon cancer, MSI-H/dMMR colon cancer, MSS/pMMR rectal cancer, and MSI-H/dMMR rectal cancer, as illustrated in Figure 1. Finally, we highlight circulating tumor DNA (ctDNA)-based minimal residual disease (MRD) assessment as a cross-cutting concept and discuss future directions for risk-adapted treatment escalation and de-escalation. The overall framework is summarized in Table 1.

## 2. MSS/pMMR Colon Cancer—Perioperative Chemotherapy

In MSS/pMMR colon cancer—the dominant molecular subtype—the principal therapeutic paradigm has been the stepwise optimization of cytotoxic chemotherapy in the postoperative setting, evolving from uniform 6-month fluoropyrimidine-based regimens toward risk-stratified duration of oxaliplatin-based therapy, as exemplified by the IDEA collaboration [4]. More recently, the FOxTROT trial has provided the first phase III evidence that neoadjuvant chemotherapy can improve outcomes in operable disease, with benefit largely confined to pMMR tumors [14]. Current frontiers are shifting from cytotoxic uniformity toward biology-guided treatment intensity, with ongoing trials evaluating the integration of immune checkpoint blockade into chemotherapy backbones in pMMR populations [19].

### 2.1. Establishment of Adjuvant Chemotherapy

From the late 1950s through the mid-1970s, adjuvant trials of single agents such as thiotepa, floxuridine (FUDR), and fluorouracil (5-FU) monotherapy largely failed to demonstrate a clear benefit [43]. The first positive signal came from NSABP C-01, in which the MOF regimen (methyl-CCNU, vincristine, and 5-FU) significantly improved disease-free survival (DFS) and improved overall survival (OS) over surgery alone [44], followed by Intergroup 0035, which established adjuvant 5-FU plus levamisole for node-positive colon cancer and led to its endorsement as standard therapy for stage III disease by the NIH Consensus Conference [45,46]. The backbone was then refined by leucovorin modulation: 5-FU/leucovorin (5-FU/LV) proved superior to MOF- and levamisole-based regimens (NSABP C-03 and C-04) [47,48], and INT-0089 established that approximately 6 months of 5-FU/LV-based therapy was sufficient, with no additional benefit from longer treatment [49]. Collectively, these trials established fluoropyrimidine-based adjuvant chemotherapy as the standard foundation upon which oxaliplatin-based intensification was subsequently built.

### 2.2. Transition to Oxaliplatin-Based Combination Therapy

In the early 2000s, the introduction of the platinum agent oxaliplatin markedly improved outcomes. The MOSAIC trial randomized patients with stage II–III colon cancer to FOLFOX4 versus 5-FU/LV and demonstrated significantly improved disease-free survival, with an overall survival benefit subsequently confirmed in the stage III subgroup, establishing FOLFOX as a standard adjuvant treatment option [2,12]. In stage II colon cancer, no statistically significant survival benefit was demonstrated overall; however, a trend toward improved DFS was observed in high-risk subgroups, reinforcing the importance of risk stratification [12].

In contrast, adding irinotecan to 5-FU/LV provided no significant survival benefit and increased toxicity across multiple phase III trials (CALGB 89803, PETACC-3, and ACCORD02); irinotecan-containing adjuvant regimens are therefore not recommended for colon cancer [50,51,52].

The benefit of adding oxaliplatin in older patients remains debated: an ACCENT pooled analysis found no significant DFS or OS benefit in patients aged 70 years or older, while oral fluoropyrimidine monotherapy retained efficacy regardless of age [53]. A larger ACCENT/IDEA analysis (n = 17,909) subsequently showed no significant difference in time to recurrence between age groups after accounting for the high competing risk of non-cancer-related death, suggesting that trial-fit older patients may derive a recurrence-preventing benefit from oxaliplatin comparable to that in younger patients [54].

### 2.3. Adoption of Oral Fluoropyrimidines

Oral fluoropyrimidines were introduced to reduce the burden of intravenous therapy. UFT plus leucovorin (UFT/LV) achieved outcomes comparable to intravenous 5-FU/LV (NSABP C-06; JCOG0205 in patients undergoing Japanese D2/D3 dissection) [55,56], and capecitabine monotherapy proved at least equivalent to 5-FU/LV in stage III disease (X-ACT) [57]. The addition of oxaliplatin as XELOX (capecitabine plus oxaliplatin) demonstrated DFS superiority over bolus 5-FU/LV (NO16968/XELOXA) [3], so that both infusional FOLFOX and oral-based XELOX became standard adjuvant options. S-1 was non-inferior to UFT/LV (ACTS-CC) [58] but failed to demonstrate non-inferiority to capecitabine (JCOG0910) [59]; capecitabine is therefore generally preferred, although S-1 remains a listed adjuvant fluoropyrimidine option in Japan [60].

### 2.4. Challenges and Limitations of Molecularly Targeted Agents in Adjuvant Setting

Although molecularly targeted agents—the anti-angiogenic antibody bevacizumab (NO16966) [61] and anti-EGFR antibodies in *KRAS* wild-type disease (OPUS, CRYSTAL, PRIME) [62,63,64]—became established in the metastatic setting, their translation to the adjuvant setting was uniformly disappointing. Adding bevacizumab to standard adjuvant chemotherapy failed to improve DFS and suggested a potential detrimental effect on OS (NSABP C-08, AVANT) [65,66], and adding anti-EGFR antibodies to adjuvant chemotherapy in resected stage III *KRAS* wild-type colon cancer conferred no incremental benefit (N0147, PETACC-8) [67,68]. Neither class is recommended for adjuvant therapy in current guidelines [60].

### 2.5. Optimization of Adjuvant Duration: Risk Stratification and the IDEA Collaboration

Although oxaliplatin-based adjuvant chemotherapy became established as a standard treatment, cumulative peripheral neuropathy and associated deterioration in quality of life remained a major concern. To address this issue, the IDEA collaboration prospectively pooled individual patient data from six phase III trials worldwide (n = 12,834) to compare 3 versus 6 months of adjuvant FOLFOX or CAPOX in stage III colon cancer, with 3-year DFS as the primary endpoint [4]. Non-inferiority of the 3-month regimen was not confirmed in the pooled population, but preplanned subgroup analyses revealed a clinically important interaction with both regimen and risk category. Three months of CAPOX was non-inferior to 6 months (HR 0.95, 95% CI 0.85–1.06), whereas 3 months of FOLFOX was inferior (HR 1.16, 95% CI 1.06–1.26; *p* = 0.001). The shorter duration was likewise acceptable in low-risk disease (T1–3/N1; HR 1.01, 95% CI 0.90–1.12) but not in high-risk disease (T4 or N2), in which 6 months remained superior (HR 1.12, 95% CI 1.03–1.23; *p* = 0.01) [4]. These findings reinforced the importance of recurrence-risk stratification when optimizing adjuvant treatment duration, and 3 months of CAPOX became a clinically acceptable option for low-risk disease.

### 2.6. Paradigm Shift Toward Perioperative and Neoadjuvant Strategies

In several malignancies, perioperative chemotherapy that includes neoadjuvant treatment has become standard; however, for colon cancer, postoperative adjuvant chemotherapy has historically remained the mainstay. Building on its pilot phase [13], the FOxTROT trial evaluated a perioperative approach for radiologically staged locally advanced operable colon cancer (T3–4, N0–2, M0), comprising 6 weeks of neoadjuvant oxaliplatin-based chemotherapy plus 18 weeks of postoperative chemotherapy, compared with the standard 24 weeks of postoperative chemotherapy alone, involving 1053 patients [14]. This perioperative strategy significantly reduced residual or recurrent disease within 2 years and improved the R0 resection rate, although the improvement in survival did not reach statistical significance [14]. Notably, the benefit was highly dependent on MMR status: a significant risk reduction was confined to pMMR tumors (RR 0.69, 95% CI 0.50–0.97; *p* = 0.043), whereas dMMR tumors derived no apparent benefit (RR 0.86, 95% CI 0.42–1.76; *p* = 0.68). Adding panitumumab for *RAS* wild-type tumors did not enhance efficacy [14]. These findings suggest that neoadjuvant chemotherapy may represent a new treatment option for pMMR locally advanced colon cancer; however, further prospective validation, particularly regarding careful patient selection using advanced imaging and biomarkers, is required before adopting it as the preferred standard of care over upfront surgical resection. Current guideline support is narrow: the NCCN Guidelines recommend preoperative systemic therapy only for clinical T4b or bulky nodal disease [24], while its broader use in the radiologically staged T3–4 population rests on the positive phase III FOxTROT trial, which has immature overall survival data. It therefore remains an emerging option rather than an established standard. Building on FOxTROT, successor phase III trials (FOxTROT-2 and FOxTROT-3) are extending neoadjuvant chemotherapy to older or frail patients and to triplet regimens [69], while trial eligibility is increasingly biomarker-directed, with contemporary trials restricting neoadjuvant chemotherapy to pMMR/MSS tumors [70] and redirecting dMMR tumors to immunotherapy [10,15].

In addition, for high-risk disease such as T4 or N2 tumors, the need for more intensive perioperative therapy has been explored. A phase II trial evaluating FOLFOXIRI as neoadjuvant chemotherapy reported substantial tumor regression [71]. To further determine the optimal intensity of preoperative treatment for this high-risk population, the randomized phase II JCOG2006 trial comparing neoadjuvant mFOLFOX6 (doublet) versus FOLFOXIRI (triplet) in patients with resectable locally advanced colon cancer is currently ongoing [72].

**Figure 2:**
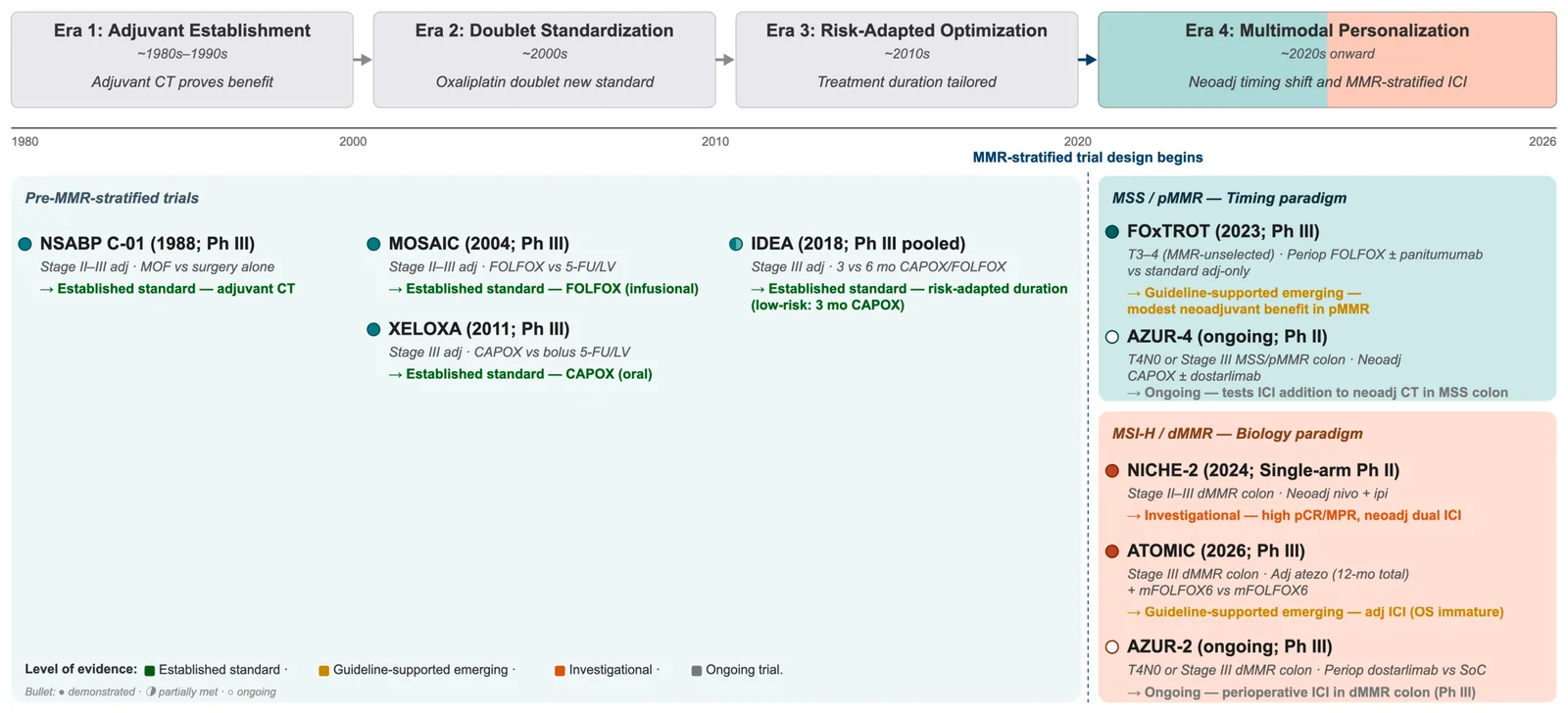
Paradigm shifts in colon cancer perioperative therapy, from cytotoxic adjuvant uniformity toward biology-driven personalization.

Furthermore, while MSS/pMMR colon cancers have historically been poorly responsive to immune checkpoint blockade, emerging strategies are attempting to overcome this resistance by combining immune checkpoint inhibitors (ICIs) with chemotherapy. The ongoing phase II AZUR-4 trial (NCT06567782) is evaluating the addition of dostarlimab, a PD-1 inhibitor, to neoadjuvant CAPEOX in patients with resectable T4N0 or stage III MSS/pMMR colon cancer, with results awaited [19]. Paradigm shifts in colon cancer perioperative therapy, from cytotoxic adjuvant uniformity toward biology-driven personalization, are illustrated in Figure 2, and pivotal adjuvant and perioperative chemotherapy trials in MSS/pMMR colon cancer are summarized in Table 2.

**Table 2:** Pivotal Trials of Perioperative Therapy in MSS/pMMR Colon Cancer.

Trial (Year)	N	Setting	Intervention vs. Comparator	Primary Endpoint	Key Result	Reference	Level of Evidence ‡
NSABP C-01 (1988; Ph III)	1166	Adjuvant, stage II–III (Dukes B–C)	MOF (methyl-CCNU + vincristine + 5-FU) vs. surgery alone	DFS; OS	DFS cumulative OR 1.29, 95% CI 1.03–1.61; *p* = 0.02; OS cumulative OR 1.31, 95% CI 1.02–1.68; *p* = 0.05	[44]	Historical (superseded)
MOSAIC (2004; Ph III)	2246	Adjuvant, stage II–III	FOLFOX4 vs. 5-FU/LV	3 yr DFS	78.2% vs. 72.9%; HR 0.77, 95% CI 0.65–0.91; *p* = 0.002	[2]	Established standard
— MOSAIC long-term (2009; Ph III)	1347 (stage III subgroup)	Adjuvant, stage III	FOLFOX4 vs. 5-FU/LV	6 yr OS	72.9% vs. 68.7%; HR 0.80, 95% CI 0.65–0.97; *p* = 0.023	[12]	Established standard
X-ACT (2005; Ph III)	1987	Adjuvant, stage III	Capecitabine vs. bolus 5-FU/LV (Mayo Clinic regimen)	3 yr DFS (non-inferiority)	64.2% vs. 60.6%; HR 0.87, 95% CI 0.75–1.00; *p* < 0.001 (NI met)	[57]	Established standard
NO16968/XELOXA (2011; Ph III)	1886	Adjuvant, stage III	XELOX vs. bolus 5-FU/LV	3 yr DFS	70.9% vs. 66.5%; HR 0.80, 95% CI 0.69–0.93; *p* = 0.0045	[3]	Established standard
IDEA (2018; Ph III pooled)—overall	12,834	Adjuvant, stage III	3 mo vs. 6 mo FOLFOX/CAPOX	3 yr DFS (non-inferiority)	74.6% vs. 75.5%; HR 1.07, 95% CI 1.00–1.15; *p* = 0.11 (NI not met)	[4]	Established standard
— CAPOX subgroup	5071	Adjuvant, stage III	3 mo vs. 6 mo CAPOX	3 yr DFS (non-inferiority)	75.9% vs. 74.8%; HR 0.95, 95% CI 0.85–1.06 (NI met)	[4]	Established standard
— FOLFOX subgroup	7763	Adjuvant, stage III	3 mo vs. 6 mo FOLFOX	3 yr DFS (non-inferiority)	73.6% vs. 76.0%; HR 1.16, 95% CI 1.06–1.26; *p* = 0.001 (inferior)	[4]	Established standard
FOxTROT (2023; Ph III)	1053	Neoadjuvant, T3–4, N0–2, M0	Neoadjuvant + adj vs. adj alone	2 yr residual/recurrent disease	16.9% vs. 21.5%; RR 0.72, 95% CI 0.54–0.98; *p* = 0.037	[13,14]	Guideline-supported emerging (cT4b or bulky nodal; pMMR) [24]
Gallois (ACCENT/IDEA, 2024; pooled analysis)	17,909	Adjuvant, stage III	Oxaliplatin-based therapy, age ≥70 vs. <70	3 yr TTR	76% vs. 77% (unadjusted *p* = 0.002); adjusted HR 1.05, 95% CI 0.98–1.12; *p* = 0.18	[54]	Supportive (pooled analysis)
CHALLENGE (2025; Ph III)	889	Post-adjuvant, resected stage III or high-risk stage II	Structured exercise vs. health education	DFS	5 yr DFS 80.3% vs. 73.9%; HR 0.72, 95% CI 0.55–0.94; *p* = 0.02; 8 yr OS 90.3% vs. 83.2%; HR 0.63, 95% CI 0.43–0.94	[73,74]	Established standard (guideline-endorsed exercise adjunct)
**— Ongoing trials —**							
JCOG2006 (Randomized Ph II)	86 (planned)	Neoadjuvant, resectable locally advanced colon (high-risk)	mFOLFOX6 (doublet) vs. FOLFOXIRI (triplet)	Tumor regression score 0–2 rate (central pathological review)	Ongoing	[72]	Ongoing
AZUR-4 (NCT06567782; Ph II)	120 (planned; 3:1)	Neoadjuvant, T4N0 or stage III MSS/pMMR	Dostarlimab + CAPEOX vs. CAPEOX	Major pathological response (local assessment); safety	Ongoing	[19]	Ongoing

*Abbreviations:* adj, adjuvant chemotherapy; CAPEOX/CAPOX, capecitabine plus oxaliplatin; CI, confidence interval; DFS, disease-free survival; 5-FU, 5-fluorouracil; FOLFOX, infusional fluorouracil/leucovorin/oxaliplatin; FOLFOXIRI, fluorouracil/leucovorin/oxaliplatin/irinotecan; HR, hazard ratio; LV, leucovorin; OR, odds ratio; mFOLFOX6, modified FOLFOX6; MSS, microsatellite stable; NI, non-inferiority; OS, overall survival; pMMR, proficient mismatch repair; RR, rate ratio; TTR, time to recurrence; XELOX, capecitabine plus oxaliplatin. *Note:* Ongoing trials are listed with NCT registry identifiers where available. ‡ Evidence-level classification as defined in the footnote to Table 1.

### 2.7. Structured Exercise as an Adjunct to Adjuvant Therapy

Beyond systemic therapy, the phase III CHALLENGE trial provided the first randomized evidence that structured exercise improves survival after resection. A 3-year structured exercise program initiated after adjuvant chemotherapy for stage III or high-risk stage II colon cancer significantly prolonged both disease-free and overall survival, and an ESMO Clinical Practice Guideline express update now endorses structured physical exercise as an adjunct in localized colon cancer [73,74].

## 3. MSI-H/dMMR Colon Cancer

In MSI-H/dMMR colon cancer, the dominant paradigm shift is the integration of ICI therapy into perioperative management, reflecting the exceptional immunogenicity of this subtype. Two distinct pivotal strategies have emerged: adjuvant ICI plus chemotherapy, exemplified by the phase III ATOMIC trial [17], and short-course neoadjuvant dual checkpoint blockade, exemplified by the NICHE-2 trial [10,15]. Both have demonstrated substantial clinical activity, positioning immunotherapy as a central and rapidly emerging therapeutic principle in this subtype, although the maturity of the supporting evidence differs between the guideline-endorsed but still-maturing adjuvant strategy and the still-investigational neoadjuvant approaches. Growing interest now centers on treatment de-escalation and selective non-operative management for highly responsive patients.

### 3.1. Molecular Biological Background of MSI-H/dMMR Colon Cancer

MSI-H or dMMR status defines a clinically and biologically distinct subtype of CRC whose frequency declines with advancing stage, occurring in approximately 15–18% of stage II and 9–10% of stage III colon cancers and falling to approximately 4–5% in metastatic colorectal cancers [42]. In sporadic cases, MSI-H tumors arise predominantly through epigenetic silencing of *MLH1* via promoter hypermethylation, while in Lynch syndrome they result from germline mutations in *MLH1*, *MSH2*, *MSH6*, or *PMS2* [75]. Lynch syndrome accounts for approximately 2–4% of all colorectal cancers, and high-penetrance *MLH1* or *MSH2* carriers face a substantially elevated cumulative colorectal cancer risk by age 70 [76]. Approximately half of sporadic MSI-H colorectal cancers harbor *BRAF* V600E mutations, typically arising in the context of the CpG island methylator phenotype (CIMP), which is associated with *MLH1* promoter hypermethylation and consequent MMR inactivation [75]. Notably, while *BRAF* V600E is a strong indicator of poor prognosis in MSS tumors, recent large pooled analyses demonstrate that it does not significantly alter the prognosis within the MSI-H subgroup [42].

The defining molecular feature of dMMR tumors is a markedly elevated somatic mutation rate, particularly insertion-deletion (indel) mutations in repetitive DNA sequences (microsatellites). The resulting frameshift mutations generate a dense repertoire of tumor-specific neoantigens—frameshift peptides—that are structurally foreign to the host immune system. Notably, within dMMR CRC tumors, the total number of frameshift mutations correlates directly with the density of intratumoral CD8+ T-cell infiltration [77]. These features collectively render dMMR tumors highly immunogenic: a meta-analysis confirmed that high tumor-infiltrating lymphocyte density—markedly enriched in MSI-H tumors—is associated with improved overall survival (HR 0.65, 95% CI 0.54–0.77; *p* < 0.01) [78]. Accordingly, MSI-H/dMMR is an independent favorable prognostic marker in resectable colon cancer, with significantly better OS than MSS/pMMR tumors in a large systematic review (HR 0.65, 95% CI 0.59–0.71) [79].

### 3.2. Limited Benefit of Conventional Adjuvant Chemotherapy in dMMR Tumors

Pooled analyses from multiple randomized trials have demonstrated limited efficacy of fluoropyrimidine-based adjuvant chemotherapy in dMMR colon cancer, with effects varying by stage and regimen. The biological basis for this resistance lies in the critical role of mismatch recognition in mediating 5-FU cytotoxicity: in pMMR tumors, the hMutSα complex recognizes 5-FU incorporated into DNA and initiates apoptosis, whereas this recognition mechanism is absent in dMMR tumors, resulting in de facto drug resistance [80]. For stage III disease, fluoropyrimidine monotherapy has been reported to show no significant efficacy in dMMR tumors (DFS HR 1.01, 95% CI 0.41–2.51; *p* = 0.98) [81]. Regarding oxaliplatin-containing regimens, a pooled analysis of two randomized trials from the ACCENT database demonstrated that adding oxaliplatin to fluoropyrimidine significantly improved both OS (HR 0.52, 95% CI 0.28–0.93) and DFS (HR 0.47, 95% CI 0.27–0.82) in dMMR stage III patients compared with fluoropyrimidine alone [42]. Consequently, when adjuvant chemotherapy is indicated for stage III disease, major clinical guidelines universally recommend oxaliplatin-containing regimens [24,60,82]. These estimates should be interpreted with caution, however, because the test for interaction was not statistically significant (*p* = 0.11) and the benefit relative to pMMR tumors was confined to N1 disease, with no comparable difference in N2 disease [42].

For stage II disease, fluoropyrimidine monotherapy not only lacks efficacy but may worsen outcomes. Two pooled analyses of randomized trials both reported worse overall survival with fluorouracil-based adjuvant therapy than with surgery alone in stage II MSI-H/dMMR disease, reaching statistical significance in the larger analysis (HR 2.95, 95% CI 1.02–8.54; *p* = 0.04) [81,83]. Based on these findings, major clinical guidelines worldwide consistently recommend against adjuvant fluoropyrimidine monotherapy for dMMR stage II colon cancer [24,60,82,84]. When adjuvant chemotherapy is considered for high-risk dMMR stage II disease, both the ASCO and NCCN guidelines recommend oxaliplatin-containing regimens over fluoropyrimidine monotherapy [24,84].

### 3.3. Immune Checkpoint Inhibition in dMMR Colon Cancer: From Metastatic to Perioperative Settings

Biologically, dMMR tumors are characterized by high neoantigen burden, abundant intratumoral CD8+ T-cell infiltration, and an inflammatory tumor microenvironment marked by upregulation of inhibitory checkpoint molecules including programmed cell death protein 1 (PD-1), programmed death-ligand 1 (PD-L1), cytotoxic T-lymphocyte-associated antigen 4 (CTLA-4), and lymphocyte-activation gene 3 (LAG-3) [77]. This state of “exhausted” antitumor immunity is reversible upon checkpoint blockade, providing the biological rationale for the striking clinical responses observed with ICIs in dMMR colon cancer [85,86].

The efficacy of ICIs in dMMR colon cancer was first established in the metastatic setting. Pembrolizumab demonstrated significant superiority over chemotherapy as first-line treatment for MSI-H/dMMR metastatic CRC in the KEYNOTE-177 trial, with a durable progression-free and overall survival benefit at long-term follow-up despite a high crossover rate to PD-(L)1 inhibitors in the chemotherapy arm [85,87]. In the CheckMate 8HW trial, nivolumab plus ipilimumab demonstrated significant superiority over chemotherapy in the first-line setting, and across all lines of therapy yielded a higher response rate and longer progression-free survival (PFS) than nivolumab monotherapy, supporting dual-checkpoint blockade as a standard-of-care option [86]. Both regimens are now used as standard first-line therapies for MSI-H/dMMR metastatic CRC. Early-stage colon cancer presents a more immunologically favorable setting compared with metastatic disease—characterized by higher T-cell infiltration, lower systemic immunosuppression, the absence of visceral metastases, and lower tumor burden—raising the hypothesis that perioperative ICIs could achieve even greater efficacy in the non-metastatic setting [9].

Evidence for ICIs in the adjuvant setting was substantially advanced by the phase III ATOMIC trial. This randomized trial enrolled 712 patients with resected stage III dMMR colon cancer and compared mFOLFOX6 alone with mFOLFOX6 plus atezolizumab (anti-PD-L1), followed by atezolizumab monotherapy (12 months of atezolizumab in total). The primary endpoint of DFS was met, with an approximate halving of the hazard of recurrence or death, supporting adjuvant atezolizumab plus mFOLFOX6 as an emerging, guideline-endorsed option rather than a definitively established standard for resected stage III dMMR colon cancer [17]. This cautious framing reflects unresolved issues: overall survival remains immature, with no significant difference between the arms (HR 0.90, 95% CI 0.55–1.47; *p* = 0.68) at a median follow-up of 45.8 months, and optimal patient selection, treatment duration, toxicity, the sequencing of adjuvant after neoadjuvant immunotherapy, and generalizability beyond the trial population all remain undefined. These results have been rapidly incorporated into clinical guidelines: the NCCN Guidelines (Version 1.2026) now designate FOLFOX plus atezolizumab as a “Preferred” adjuvant regimen for resected MSI-H/dMMR stage III colon cancer, applicable to both low-risk (T1–3, N1) and high-risk (T4 or N2) subgroups [24]. Notably, the guidelines include a caveat that the role of adjuvant immunotherapy following neoadjuvant immunotherapy remains undefined, underscoring the need for prospective data in this emerging population.

While the ATOMIC trial demonstrated a role for adjuvant immunotherapy in dMMR colon cancer, evidence from other solid tumors—particularly melanoma—suggests that a neoadjuvant approach may be advantageous relative to adjuvant therapy alone. Importantly, both phase II and pivotal phase III randomized trials in resectable melanoma have confirmed that neoadjuvant immune checkpoint blockade significantly prolongs event-free survival (EFS) compared with standard adjuvant therapy [88,89]. From an immunologic perspective, neoadjuvant therapy is highly appealing because the presence of the intact primary tumor provides an abundant source of neoantigens, thereby facilitating optimal immune priming and the robust expansion of tumor-specific T cells before the tumor bulk is surgically removed. On the basis of this biological rationale and cross-tumor clinical success, neoadjuvant ICI strategies were subsequently explored in dMMR colon cancer.

The potential for neoadjuvant ICIs in dMMR colon cancer was first demonstrated by the NICHE-1 trial, a phase II study in which patients with non-metastatic colon cancer received short-course preoperative ipilimumab plus nivolumab [9]. Pathological response was observed in nearly all evaluable dMMR patients, with the majority achieving a major pathological response (MPR, defined as ≤10% residual viable tumor), in striking contrast to the modest response rate among pMMR patients—demonstrating the exceptional immunosensitivity of dMMR tumors. Building on this proof-of-concept, the pivotal NICHE-2 trial enrolled 115 patients with dMMR colon cancer using the same short-course regimen, achieving a pathological response rate of 98% together with high rates of major and complete pathological response and no recurrences at the reported follow-up [10,15]. These single-arm, non-randomized data indicate that short-course neoadjuvant dual checkpoint blockade induces exceptionally high pathological response rates in resectable dMMR colon cancer; however, pathological response and short-term DFS have not yet been validated as surrogates for long-term survival in this setting, and randomized confirmation (e.g., the ongoing AZUR-2 trial) is awaited.

Parallel investigations explored alternative checkpoint combination strategies. The NICHE-3 trial evaluated neoadjuvant nivolumab plus relatlimab (a LAG-3 inhibitor) in dMMR colon cancer patients and achieved pathological response, MPR, and pCR rates broadly comparable to NICHE-2 outcomes; the grade 3–4 immune-related adverse event (irAE) rate was modestly higher than in NICHE-2 and requires further evaluation [16]. The role of single-agent PD-1 blockade was examined in the RESET-C trial, in which stage I–III dMMR colon cancer patients received single-cycle pembrolizumab prior to surgery and achieved substantial pCR and MPR rates with R0 resection in 98% of patients, supporting the activity of even abbreviated PD-1 monotherapy in this subtype [18]. Addressing whether dual blockade adds depth of response, the randomized phase Ib NEOSHOT trial demonstrated that dual checkpoint blockade (anti-CTLA-4 plus anti-PD-1) significantly improved the pathological complete response rate compared with PD-1 monotherapy in dMMR colon cancer, providing the first randomized evidence that dual blockade can enhance response depth in this setting [90].

In contrast, the FOxTROT trial demonstrated that the dMMR subgroup derived no significant benefit from neoadjuvant cytotoxic chemotherapy with fluoropyrimidine plus oxaliplatin. Moderate or greater pathological regression was substantially less frequent in dMMR than in pMMR tumors, and no significant reduction in 2-year recurrence was observed in dMMR patients despite the benefit demonstrated in the pMMR population [14], reaffirming that conventional cytotoxic chemotherapy-based neoadjuvant strategies have limited efficacy in dMMR colon cancer.

### 3.4. Toward Treatment De-Escalation and Surgery Omission

The high pathological response rates achieved in the pivotal NICHE-2 trial and corroborating short-course neoadjuvant ICI studies (NICHE-3, RESET-C) have prompted growing interest in applying NOM—an approach with a longer track record in rectal cancer—to dMMR colon cancer.

Initial proof-of-concept data for NOM were provided by a phase II trial enrolling 35 patients with locally advanced MSI-H/dMMR solid tumors, including 27 with CRC [91]. Patients received pembrolizumab for up to 16 cycles, with approximately half pursuing NOM after achieving complete response [91]. Three-year follow-up demonstrated favorable event-free and overall survival, with no significant difference between the surgical and NOM groups, supporting the feasibility of organ preservation; ctDNA dynamics following pembrolizumab were strongly predictive of outcome, with ctDNA-negative patients achieving markedly superior EFS compared with ctDNA-positive patients [92]. Further prospective evidence for NOM in non-rectal dMMR tumors was provided by Cohort 2 of the dostarlimab phase II trial [22]. Patients with stage I–III dMMR tumors received dostarlimab for 6 months, achieving substantial clinical complete response (cCR) rates—including a notably high cCR rate in the colon cancer subgroup—with favorable short-term recurrence-free survival (RFS) [22].

These findings highlight the urgency of establishing rigorous eligibility criteria for NOM, incorporating ctDNA, FDG-PET/CT, and colonoscopy in a multimodal assessment framework [16]. Colon cancer presents unique challenges compared with rectal cancer—particularly the limited accuracy of standard radiographic assessment and the inability to use digital rectal examination or pelvic MRI. Furthermore, the rationale for NOM differs fundamentally between colon and rectal cancers: because colon resection generally carries a lower risk of permanent colostomy and long-term functional impairment, the threshold for omitting surgery is significantly higher [22]. Importantly, the incidence of severe (grade 3–4) irAEs with single-cycle PD-1 monotherapy has been acceptable [18]. Nevertheless, as highlighted by the RESET-C trial, patient selection must carefully balance the risk of these potentially irreversible irAEs and residual disease against surgery-induced morbidity [18]. In particular, patients with low-risk stage I–II dMMR colon cancer have a highly favorable prognosis with surgery alone; thus, neoadjuvant ICIs and subsequent NOM may be unjustifiable due to the risk of overtreatment and irreversible irAEs [10]. Conversely, for patients with stage III or high-risk disease, the primary rationale for neoadjuvant ICIs is not organ preservation per se, but rather the critical need for highly effective systemic therapy to prevent recurrence [10]. It is primarily within this high-risk population—or among frail patients for whom surgery poses a prohibitive risk of morbidity or mortality—that the option of NOM becomes a clinically relevant consideration upon achieving a cCR. Ultimately, prospective trials are needed to define safe NOM protocols and identify the appropriate subset of patients for this approach [10,18,22].

### 3.5. Ongoing and Future Trials

The evolving landscape of perioperative immunotherapy in dMMR colon cancer has generated a robust pipeline of ongoing and planned clinical trials aimed at establishing randomized evidence, addressing the NOM question, and refining patient selection. AZUR-2 is the pivotal phase III randomized trial designed to provide confirmatory randomized evidence for perioperative dostarlimab (anti-PD-1) in dMMR colon cancer. Targeting approximately 711 patients with previously untreated T4N0 or stage III resectable dMMR colon cancer, AZUR-2 randomizes patients to perioperative dostarlimab monotherapy versus immediate surgery followed by standard of care (adjuvant chemotherapy or surveillance). The primary endpoint is EFS, with secondary endpoints including OS, pathological response assessed by residual viable tumor (RVT), and safety. The primary analysis is expected in December 2028 [23].

Pivotal neoadjuvant immunotherapy and adjuvant ICI trials in MSI-H/dMMR colon cancer are summarized in Table 3.

**Table 3:** Pivotal Trials of Perioperative Therapy in MSI-H/dMMR Colon Cancer.

Trial (Year)	N	Setting	Regimen	pCR (%)	MPR (%)	DFS/ Follow-Up	Reference	Level of Evidence ‡
NICHE-1 (2020; Open-label Ph II)	20 (dMMR cohort only) §	Neoadjuvant, non-metastatic	Ipilimumab + nivolumab (short-course)	60% (12/20)	95% (19/20); 100% pathological response	No recurrence reported	[9]	Investigational (proof-of-concept)
NICHE-2 (2024; Single-arm Ph II)	115	Neoadjuvant, stage II–III colon	Ipilimumab + nivolumab (short-course)	68% (95% CI 58–76)	95% (95% CI 89–98)	No recurrences at median 26.2 mo; 3 yr DFS 100% (ESMO 2024)	[10,15]	Investigational (single-arm)
NICHE-3 (2024; Single-arm Ph II)	59	Neoadjuvant, colon	Nivolumab + relatlimab (anti-LAG-3)	68% (95% CI 54–79)	92% (95% CI 81–97)	Pathological response 97% (95% CI 88–100)	[16]	Investigational (single-arm)
Ludford NOM cohort (pembrolizumab, 2023–2025; Ph II) ¶	35 total (27 CRC subset)	Neoadjuvant, stage I–III solid tumors	Pembrolizumab up to 16 cycles	65% overall (79% CRC) among operated	—	3 yr EFS 80% (95% CI 66–93); 3 yr OS 94% (95% CI 86–100)	[91,92]	Investigational (single-arm, NOM)
Cercek dostarlimab Cohort 2 (non-rectal, 2025; Ph II)	54 completed	Neoadjuvant, stage I–III non-rectal	Dostarlimab 6 mo	—	—	cCR 65% (35/54; colon 81%); 2 yr RFS 85% (95% CI 70–100) at median 14.9 mo	[22]	Investigational (single-arm, NOM)
NEOSHOT (2025; Randomized Ph Ib) †	—	Neoadjuvant, colon (randomized phase Ib)	IBI310 (anti-CTLA-4) + sintilimab vs. sintilimab alone	78.4% vs. 46.7%; *p* = 0.0015	—	—	[90]	Investigational (randomized Ph Ib)
ATOMIC (2026; Ph III)	712	Adjuvant, stage III	mFOLFOX6 ± atezolizumab (12 mo)	—	—	3 yr DFS 86.3% vs. 76.2%; HR 0.50, 95% CI 0.35–0.73; *p* = 0.0002	[17]	Guideline-supported emerging (OS immature)
RESET-C (2026; Ph II)	84 evaluable (85 enrolled)	Neoadjuvant, stage I–III	Single-cycle pembrolizumab	44% (37/84; 95% CI 33–55)	57% (48/84; 95% CI 46–68)	R0 resection in 98% (82/84; 95% CI 92–100)	[18]	Investigational (single-arm)
**— Ongoing trials —**								
AZUR-2 (NCT05855200; Ph III)	711 (planned)	Perioperative, T4N0 or stage III colon	Perioperative dostarlimab vs. immediate surgery + standard of care	TBD	TBD	Primary endpoint EFS; primary analysis expected December 2028	[23]	Ongoing

*Note:* Cercek dostarlimab Cohort 2 is the non-rectal cohort of the same phase II study whose rectal cohort is shown in the MSI-H/dMMR rectal cancer table below; the two rows therefore report different cohorts of one trial [22]. ¶ Ludford NOM cohort: phase II trial of pembrolizumab in MSI-H/dMMR solid tumors (35 patients, 27 with CRC) with 51.4% electing NOM; 3 yr follow-up reported [92]. Included as the first dedicated NOM-feasibility cohort in non-rectal dMMR disease. † NEOSHOT enrolled dMMR colon cancer patients in a randomized phase Ib design and is included as the first randomized comparator demonstrating dual vs. single-agent ICI superiority in this disease. § NICHE-1 originally enrolled 40 patients (21 dMMR and 20 pMMR tumors; one patient had both); only the dMMR cohort data are shown in this table as the table focuses on MSI-H/dMMR trials. *Abbreviations:* cCR, clinical complete response; CI, confidence interval; CRC, colorectal cancer; CTLA-4, cytotoxic T-lymphocyte-associated protein 4; DFS, disease-free survival; dMMR, deficient mismatch repair; EFS, event-free survival; HR, hazard ratio; ICI, immune checkpoint inhibitor; LAG-3, lymphocyte-activation gene 3; mFOLFOX6, modified FOLFOX6; MPR, major pathological response; MSI-H, microsatellite instability-high; NOM, non-operative management; OS, overall survival; pCR, pathological complete response; pMMR, proficient mismatch repair; R0, microscopically margin-negative resection; RFS, recurrence-free survival; TBD, to be determined. ‡ Evidence-level classification as defined in the footnote to Table 1.

## 4. MSS/pMMR Rectal Cancer

In MSS/pMMR rectal cancer, organ preservation has emerged as a primary therapeutic goal that fundamentally distinguishes management from the predominantly adjuvant paradigm of colon cancer. TNT has reshaped the standard of care for high-risk locally advanced disease, with pivotal evidence from RAPIDO and PRODIGE 23 [5,6], while the OPRA trial established the consolidation sequence as preferred when watch-and-wait organ preservation is the goal [7]. For lower-risk disease, PROSPECT provided pivotal evidence that selective omission of pelvic radiotherapy is oncologically safe [8]. Current frontiers include response-adapted sequencing and integration of immune checkpoint blockade into neoadjuvant strategies.

While molecular profiling—particularly MMR status—increasingly informs perioperative decisions across both colon and rectal cancer, the management of rectal cancer is additionally dictated by unique anatomical constraints that necessitate a fundamentally different therapeutic framework, including organ preservation.

### 4.1. From Postoperative to Preoperative Chemoradiotherapy (CRT): Historical Development and Anatomical Rationale

The perioperative management of rectal cancer diverges from that of colon cancer because of unique anatomical constraints. The rectum is confined within the bony pelvis, with narrow fascial boundaries that constrain resection margins and distal clearance owing to its proximity to the anal sphincter complex [93]. Before standardized total mesorectal excision (TME), local recurrence after surgery alone was common [94]. TME—complete excision of the mesorectum [95]—established the circumferential resection margin (CRM) as the central surgical quality benchmark, with a positive CRM (tumor within 1 mm) serving as the dominant pathological determinant of local recurrence [96], and substantially reduced local recurrence even without preoperative radiotherapy [97]. Because radical resection of the lower rectum also carries a substantial risk of permanent colostomy and urinary and sexual dysfunction [98], reducing both locoregional recurrence and surgical morbidity became a persistent dual imperative driving the multimodal paradigm described in this section [93,98].

Randomized trials progressively established combined-modality therapy. Postoperative radiotherapy reduced locoregional recurrence without improving survival (NSABP R-01) [99], whereas adding concurrent chemotherapy (postoperative chemoradiotherapy, CRT) reduced both recurrence and mortality (Gastrointestinal Tumor Study Group 7175; North Central Cancer Treatment Group Intergroup trial), leading the 1990 NIH Consensus Conference to recommend postoperative CRT for stage II–III disease [46,100,101]. Because surgical trauma impairs the radiosensitivity and tolerability of postoperative treatment, attention shifted to the preoperative window. The Swedish Rectal Cancer Trial first showed that preoperative radiotherapy reduces local recurrence and improves OS [94], and the Dutch TME trial confirmed a reduced 2-year local recurrence rate when preoperative short-course radiotherapy (SCRT; 25 Gy in five fractions) preceded TME—although distant metastases remained the predominant pattern of failure, underscoring the need for effective systemic therapy [97].

Two randomized trials (the Polish trial and Trans-Tasman Radiation Oncology Group 01.04) established clinical equipoise between SCRT and long-course chemoradiotherapy (LC-CRT) for overall oncological outcomes, with SCRT causing markedly less acute toxicity but yielding less tumor downstaging and a lower pathological complete response rate than LC-CRT—a limitation that later motivated the addition of interval systemic chemotherapy in SCRT-based TNT approaches [102,103]. The pivotal CAO/ARO/AIO-94 trial then established preoperative CRT as the global standard for locally advanced rectal cancer (LARC), significantly reducing the 5-year local recurrence rate with lower toxicity and higher sphincter preservation in low-lying tumors compared with postoperative CRT, without an OS difference [104]. Subsequent intensification with oxaliplatin (CAO/ARO/AIO-04) significantly improved both the pCR rate and 3-year DFS, providing an additional multimodal option [105].

Two limitations nonetheless persisted. First, improved locoregional control did not consistently translate into a survival benefit, as illustrated by EORTC 22921, in which adding fluoropyrimidine to preoperative radiotherapy reduced local recurrence without an OS benefit [106]. Second, compliance with postoperative adjuvant chemotherapy was consistently poor—fewer than half of patients completed the planned regimen—limiting the systemic exposure needed to address distant metastases, which remained the dominant cause of cancer-related mortality [106]. Attempts to address this through postoperative adjuvant chemotherapy were largely unsuccessful, with only a possible benefit from oxaliplatin-containing regimens in residual node-positive disease [107,108,109]. This failure provided the principal rationale for TNT: by integrating all planned systemic chemotherapy into the preoperative window, before surgical functional impairment, TNT aimed to improve systemic exposure and tumor response—the central hypothesis of the landmark trials described in the following section.

### 4.2. The Rise of Total Neoadjuvant Therapy

Key TNT trials in locally advanced rectal cancer (phase III: Polish II, RAPIDO, PRODIGE 23, STELLAR; randomized phase II: OPRA) are summarized in Table 4A.

TNT integrates all planned systemic chemotherapy into the preoperative window to improve adherence, enhance tumor response, and address the systemic disease burden that conventional adjuvant strategies had failed to control. Conceptual groundwork came from Stockholm III, which demonstrated that delaying surgery after SCRT was oncologically non-inferior to immediate surgery and significantly reduced postoperative complications, establishing that this interval could be safely exploited for consolidation systemic therapy in SCRT-based TNT strategies [110].

Polish II then provided clinical proof of concept, showing that SCRT followed by FOLFOX4 consolidation was feasible and tolerable in high-risk disease, with long-term oncological outcomes equivalent to long-course oxaliplatin-containing CRT, thereby establishing the precedent for the larger phase III trials that followed [111,112].

RAPIDO was the first phase III trial to demonstrate a significant benefit with a TNT strategy, enrolling 920 patients with high-risk LARC—defined as cT4a/b, extramural vascular invasion (EMVI) positivity, cN2, threatened mesorectal fascia (MRF), or enlarged lateral lymph nodes. SCRT (25 Gy in five fractions) followed by 6 cycles of CAPOX or 9 cycles of FOLFOX4 consolidation chemotherapy and TME was compared with the standard arm of long-course capecitabine-based CRT (50.4 Gy) followed by TME and adjuvant CAPOX or FOLFOX4 [5]. The primary endpoint, 3-year disease-related treatment failure (locoregional failure, distant metastases, new primary colorectal tumor, or treatment-related death), was significantly lower in the experimental arm, and the pCR rate was significantly higher. Three-year OS did not differ between the arms, and 5-year locoregional recurrence was significantly higher with SCRT-based TNT than with the standard arm (10% vs. 6%, *p* = 0.027) [21]. This finding has informed subsequent guideline recommendations favoring long-course CRT when organ preservation or optimal local control is the treatment goal [113].

**Table 4:** (**A**) Pivotal Trials of Perioperative Therapy in MSS/pMMR Rectal Cancer. (**B**) Pivotal Trials of Perioperative Immunotherapy in MSS/pMMR Rectal Cancer.

**(A)**								
**Trial (Year)**	**N**	**Setting**	**Strategy/** **Design**	**Primary Endpoint**	**Key Result**	**Organ Preservation Rate**	**Reference**	**Level of Evidence ‡**
Swedish Rectal Cancer Trial (1997; Ph III)	1168	Neoadjuvant, resectable rectal	SCRT (25 Gy/5f) → surgery vs. surgery alone	5 yr OS; local recurrence	5 yr OS 58% vs. 48%; HR 0.79, 95% CI 0.66–0.92; *p* = 0.004; 5 yr LR 11% vs. 27%; *p* < 0.001	—	[94]	Historical
Dutch TME (2001; Ph III)	1861	Neoadjuvant, resectable rectal	SCRT (25 Gy/5f) → TME vs. TME alone	2 yr local recurrence	2 yr LR 2.4% vs. 8.2%; *p* < 0.001; 2 yr OS 82.0% vs. 81.8%; HR 1.02, 95% CI 0.83–1.25; *p* = 0.84	—	[97]	Historical
CAO/ARO/AIO-94 (2004; Ph III)	823	Neoadjuvant vs. adjuvant, stage II–III	Preoperative CRT vs. postoperative CRT (50.4 Gy + 5-FU)	5 yr OS	5 yr OS 76% vs. 74%; *p* = 0.80; 5 yr LR 6% vs. 13%; *p* = 0.006	—	[104]	Established standard (preop CRT)
EORTC 22921 (2006; Ph III)	1011	Perioperative, stage II–III	Preoperative RT (45 Gy) ± concurrent 5-FU/LV ± postoperative 5-FU/LV (2 × 2 factorial)	5 yr OS	5 yr OS 65.8% (preop CRT) vs. 64.8% (preop RT); HR 1.02; *p* = 0.84; 5 yr OS 67.2% vs. 63.2% (± postop chemo); HR 0.85; *p* = 0.12; 5 yr LR with any chemotherapy 7.6–9.6% vs. 17.1% (RT alone); *p* = 0.002	—	[106]	Historical
CAO/ARO/AIO-04 (2015; Ph III)	1236	Neoadjuvant, stage II–III	Preoperative fluorouracil-based CRT ± oxaliplatin	3 yr DFS	3 yr DFS 75.9% vs. 71.2%; HR 0.79, 95% CI 0.64–0.98; *p* = 0.03; pCR 17% vs. 13%; OR 1.41, 95% CI 1.03–1.94; *p* = 0.031	—	[105]	Established standard
Polish II (2016; Ph III)	515	Neoadjuvant, LARC (high-risk)	SCRT → FOLFOX × 3 vs. LC-CRT + oxaliplatin	R0 resection	3 yr OS 73% vs. 65%; HR 0.73, 95% CI 0.53–1.01; *p* = 0.046	—	[111]	Established (TNT precedent)
— Polish II long-term (2019; Ph III)	515	Neoadjuvant, LARC (high-risk)	Same	8 yr OS	8 yr OS 49% vs. 49%; HR 0.90, 95% CI 0.70–1.15; *p* = 0.38	—	[112]	Established (TNT precedent)
RAPIDO (2021; Ph III)	920	Neoadjuvant, LARC (high-risk)	SCRT → CAPOX × 6 vs. LC-CRT → adj chemo	3 yr DrTF	3 yr DrTF 23.7% vs. 30.4%; HR 0.75, 95% CI 0.60–0.95; *p* = 0.019; pCR 28% vs. 14%; OR 2.37, 95% CI 1.67–3.37; *p* < 0.0001	—	[5]	Established standard (TNT)
—RAPIDO 5 yr (2023; Ph III)	920	Neoadjuvant, LARC (high-risk)	Same	5 yr locoregional recurrence	5 yr LRR 10% vs. 6%; *p* = 0.027	—	[21]	Established standard (TNT)
PRODIGE 23 (2021; Ph III)	461	Neoadjuvant, LARC (cT3–4)	mFOLFIRINOX × 6 → CAPECRT → adj vs. CAPECRT → adj	3 yr DFS	3 yr DFS 76% vs. 69%; HR 0.69, 95% CI 0.49–0.97; *p* = 0.034; pCR 28% vs. 12%; *p* < 0.0001	—	[6]	Established standard (TNT)
— PRODIGE 23 7 yr (2024; Ph III)	461	Neoadjuvant, LARC (cT3–4)	Same	7 yr OS	7 yr OS 81.9% vs. 76.1%; RMST difference 4.37 mo, 95% CI 0.35–8.38; *p* = 0.033	—	[20]	Established standard (TNT)
STELLAR (2022; Ph III)	599	Neoadjuvant, LARC	SCRT → CAPOX × 4 vs. LC-CRT (CAPECRT)	3 yr DFS (non-inferiority)	3 yr DFS 64.5% vs. 62.3%; HR 0.883, one-sided 95% CI upper limit 1.11; *p* < 0.001 (NI met); pCR + sustained cCR 21.8% vs. 12.3%; *p* = 0.002	—	[114]	Established standard (TNT); nominal OS benefit requires confirmation
CAO/ARO/AIO-12 (2022; Randomized Ph II)	311	Neoadjuvant, LARC	Induction vs. consolidation FOLFOX with LC-CRT	pCR	pCR 25% (consolidation) vs. 17% (induction); 3 yr DFS 73% vs. 73%	—	[25]	Guideline-supported emerging (W&W sequencing)
OPRA (2022; Randomized Ph II)	324	Neoadjuvant, LARC; W&W eligible	Induction vs. consolidation CRT + chemotherapy, response-adapted	3 yr DFS (primary); TME-free survival (key secondary)	3 yr TME-free 53% (consolidation) vs. 41% (induction); *p* = 0.01; 3 yr DFS 76% vs. 76% (no significant difference)	53% vs. 41% at 3 yr; *p* = 0.01	[7]	Guideline-supported emerging (W&W)
— OPRA 5 yr (2024; Randomized Ph II)	324	Neoadjuvant, LARC; W&W eligible	Same	5 yr DFS; TME-free survival; OS	5 yr TME-free 54% vs. 39%; *p* = 0.012; 5 yr DFS 69% vs. 71%; *p* = 0.68; 5 yr OS 85% vs. 88%; *p* = 0.25	54% vs. 39% at 5 yr; *p* = 0.012	[26]	Guideline-supported emerging (W&W)
NOMINATE (2022 protocol; Randomized Ph II)	66 planned (64 enrolled)	Neoadjuvant, distal LARC; W&W eligible	Consolidation vs. sandwich TNT, NOM if cCR/near-cCR	Feasibility of TNT + NOM	NOM elected in 39% (25/64 evaluable) achieving cCR/near-cCR	NOM in 39% of evaluable	[28,29]	Investigational (W&W)
ENSEMBLE-1 (2023; Ph II)	30	Neoadjuvant, LARC (Japanese feasibility)	SCRT → CAPOX × 6	pCR	pCR 30%; treatment completion 83%	—	[115]	Investigational (feasibility)
PROSPECT (2023; Ph II/III)	1194	Neoadjuvant, cT2N+/cT3 LARC (MRF–)	FOLFOX ± selective CRT vs. LC-CRT	5 yr DFS (non-inferiority)	5 yr DFS 80.8% vs. 78.6%; HR 0.92, 90.2% CI 0.74–1.14; *p* = 0.005 (NI met); 89.6% avoided CRT	—	[8]	Established standard (RT omission)
ENSEMBLE-2 (2024; Ph II)	28 (21 resected)	Neoadjuvant, LARC (Japanese feasibility)	LC-CRT → CAPOX × 4	pCR	pCR 23.8% (5/21 resected; 90% CI 11.8–41.8); TNT completion 96.4% (27/28)	—	[116]	Investigational (feasibility)
CAO/ARO/AIO-16 (2025; Ph II)	91	Neoadjuvant, LARC; W&W eligible	LC-CRT (50.4 Gy + oxali) → FOLFOX × 3, selective W&W	cCR rate; initial OP	cCR 37%; initial OP 40%; 3 yr TME-free 21%; 3 yr OS 94%	40% initial; 21% at 3 yr	[117]	Investigational (W&W)
FOWARC 10 yr (2025; Ph III)	495	Neoadjuvant, stage II–III	mFOLFOX6 alone vs. mFOLFOX6 + RT vs. 5-FU + RT	10 yr DFS	10 yr DFS 60.5% vs. 62.6% vs. 52.5%; *p* = 0.56; 10 yr LRR 9.6% vs. 8.0% vs. 10.8%; *p* = 0.57	—	[118]	Supportive (long-term)
NO-CUT (2025; Ph II)	179	Neoadjuvant, LARC; W&W eligible	Induction CAPOX → LC-CRT, selective W&W	30-mo DRFS (cCR cohort)	cCR 26%; 30-mo DRFS 95% (W&W cohort); 2 yr rectal surgery-free 83%	83% at 2 yr (W&W cohort)	[119]	Investigational (W&W)
**— Ongoing trials —**								
Janus Rectal Cancer Trial (Alliance A022104/NRG-GI010; NCT05610163; Ph II/III)	—	Neoadjuvant, LARC	Randomized TNT: doublet (mFOLFOX6 or CAPOX) vs. triplet (mFOLFIRINOX)	cCR (phase II); DFS (phase III)	TBD	TBD	[120]	Ongoing
ENSEMBLE phase III (NCT05646511; Ph III)	608 (planned; MSS/pMMR)	Neoadjuvant, LARC	SCRT → consolidation CAPOXIRI (triplet) vs. CAPOX (doublet)	Organ preservation-adapted DFS	TBD	TBD	[121]	Ongoing
**(B)**								
**Trial (Year)**	**N**	**Setting**	**Strategy/Design**	**Primary Endpoint**	**Key Result**		**Reference**	**Level of Evidence ‡**
NRG-GI002 pembrolizumab arm (2021; Ph II)	185	Neoadjuvant, LARC	FOLFOX induction + CRT ± pembrolizumab	NAR score; pCR	NAR 11.53 vs. 14.08; *p* = 0.26; pCR 31.9% vs. 29.4%; *p* = 0.75 (primary endpoint not met)		[122]	Investigational (negative)
VOLTAGE-A pMMR cohort (2022; Ph Ib/II)	39 (37 in primary endpoint analysis)	Neoadjuvant, LARC	LC-CRT (50.4 Gy + capecitabine) → nivolumab ×5 → TME	pCR	pCR 30% (11/37)		[123]	Investigational (single-arm)
PANDORA (2023; Ph II)	60 (55 evaluable)	Neoadjuvant, LARC	LC-CRT → consolidation durvalumab ×3	pCR	pCR 34.5% (95% CI 22.2–48.6); R0 96.4%; no Gr 4–5 AEs		[124]	Investigational (single-arm)
UNION (2024; Ph III)	231	Neoadjuvant, cT3–4 or N+ LARC	SCRT → CAPOX + camrelizumab vs. LC-CRT → CAPOX	pCR	pCR 39.8% vs. 15.3%; OR 3.70, 95% CI 2.0–6.9; *p* < 0.001; Grade 3 TRAE 29.2% vs. 27.2%		[27]	Investigational (Ph III; survival pending)
POLAR-STAR (2025; Randomized Ph II)	186	Neoadjuvant, LARC (all-comer pMMR + dMMR)	CRT ± concurrent vs. sequential PD-1 blockade	pCR	pCR sequential 32.7% vs. control 14.0%; RR 2.332, 95% CI 1.106–4.916; *p* = 0.019; pCR concurrent 27.1% vs. 14.0%; *p* = 0.082		[125]	Investigational (randomized Ph II)
FIRM (2026; Ph II)	30 (28 evaluable)	Neoadjuvant, LARC (≤15 cm from anal verge)	Radiation-free regimen: mFOLFOX6 + serplulimab ×6	pCR; MPR	pCR 42.9%; MPR 67.9%; high rectal subgroup pCR 71.4%/MPR 85.7%; no anastomotic fistula		[126]	Investigational (single-arm)
**— Ongoing trials —**								
STELLAR II (2025; NCT05484024; seamless Ph II/III)	218 (phase II); 588 (phase III planned)	Neoadjuvant, LARC	SCRT → CAPOX or mFOLFOX ± sintilimab	Phase II: CR rate; phase III: 3 yr DFS	Phase II CR 45.5% (iTNT) vs. 25.0% (TNT); *p* = 0.003 (phase III transition criterion met); 3 yr DFS pending; Gr 3–4 TRAE 34.5% vs. 19.4%; *p* = 0.012		[30]	Ongoing

(**A**) *Note:* Ongoing trials are listed with NCT registry identifiers where available. TBD = to be determined. *Abbreviations:* adj, adjuvant chemotherapy; CAPECRT, capecitabine-based long-course CRT; CAPOX, capecitabine plus oxaliplatin; CAPOXIRI, capecitabine plus oxaliplatin plus irinotecan; cCR, clinical complete response; CI, confidence interval; CRT, chemoradiotherapy; DFS, disease-free survival; DrTF, disease-related treatment failure; DRFS, distant relapse-free survival; FOLFOX, infusional fluorouracil/leucovorin/oxaliplatin; 5-FU, 5-fluorouracil; HR, hazard ratio; LARC, locally advanced rectal cancer; LC-CRT, long-course chemoradiotherapy; LR, local recurrence; LRR, locoregional recurrence; LV, leucovorin; mFOLFIRINOX, modified FOLFIRINOX; mFOLFOX6, modified FOLFOX6; MSS, microsatellite stable; NI, non-inferiority; NOM, non-operative management; OP, organ preservation; OR, odds ratio; OS, overall survival; pCR, pathological complete response; pMMR, proficient mismatch repair; R0, microscopically margin-negative resection; RMST, restricted mean survival time; RT, radiotherapy; SCRT, short-course radiotherapy; TBD, to be determined; TME, total mesorectal excision; TNT, total neoadjuvant therapy; W&W, watch-and-wait. (**B**) *Note:* Ongoing trials are listed with NCT registry identifiers where available. TBD = to be determined. This table covers immune-radiation and radiation-free immunochemotherapy strategies in MSS/pMMR rectal cancer. *Abbreviations:* AE, adverse event; CAPOX, capecitabine plus oxaliplatin; CI, confidence interval; CR, complete response; CRT, chemoradiotherapy; DFS, disease-free survival; dMMR, deficient mismatch repair; FOLFOX, infusional fluorouracil/leucovorin/oxaliplatin; Gr, grade; iTNT, immunotherapy-augmented TNT; LARC, locally advanced rectal cancer; LC-CRT, long-course chemoradiotherapy; mFOLFOX6, modified FOLFOX6; MPR, major pathological response; MSS, microsatellite stable; NAR, neoadjuvant rectal score; OR, odds ratio; pCR, pathological complete response; PD-1, programmed cell death protein 1; pMMR, proficient mismatch repair; R0, microscopically margin-negative resection; RR, risk ratio; SCRT, short-course radiotherapy; TBD, to be determined; TME, total mesorectal excision; TNT, total neoadjuvant therapy; TRAE, treatment-related adverse event. ‡ Evidence-level classification as defined in the footnote to Table 1.

An alternative induction-based strategy was examined in PRODIGE 23, which enrolled 461 patients with cT3–T4 rectal cancer [6]. Randomization was to 6 cycles of modified FOLFIRINOX (mFOLFIRINOX) induction, followed by standard capecitabine-based CRT (CAPECRT), TME, and 3 months of adjuvant mFOLFOX6 or capecitabine, versus CAPECRT alone followed by TME and 6 months of adjuvant mFOLFOX6 or capecitabine. At 3-year follow-up, both DFS and the pCR rate were significantly improved with TNT. Updated 7-year results confirmed the durability of these benefits, with a significant OS gain (81.9% vs. 76.1%; *p* = 0.033)—making PRODIGE 23 the first phase III trial to demonstrate an OS benefit with TNT in LARC—together with improved DFS and metastasis-free survival [20].

STELLAR similarly established the feasibility of SCRT-based TNT in an Asian LARC population enriched for stage III disease, meeting its non-inferiority endpoint for 3-year DFS with higher complete-response rates; its nominal OS benefit, however, requires confirmation, and grade 3–5 acute toxicities were more frequent with TNT [114].

Together, the RAPIDO and PRODIGE 23 trials established TNT as the new standard for high-risk LARC [5,6]. A network meta-analysis (n = 4602) confirmed that TNT improved OS compared with both LC-CRT (HR 0.73, 95% credible interval [CrI] 0.60–0.92) and SCRT alone (HR 0.67, 95% CrI 0.47–0.95), and increased both pCR and cCR rates, without increasing toxicity or compromising treatment compliance [127]. These findings have shaped a broad consensus in international guidelines: the ESMO Clinical Practice Guideline (2025) [128], the ASCO Clinical Practice Guideline (2024) [113], and the NCCN Clinical Practice Guidelines in Oncology (2026) [129] all establish TNT as the standard of care for high-risk locally advanced rectal cancer, outlining pathways for consolidation or induction chemotherapy.

Regional surgical philosophies also influence the adoption of perioperative strategies. Clinical practice in Japan follows a historically distinct, surgery-first paradigm: the JSCCR Guidelines (2024) center on autonomic nerve-preserving TME combined with extensive lateral lymph node dissection (LLND)—achieving excellent locoregional outcomes without routine preoperative radiotherapy—and classify preoperative CRT as only weakly recommended and TNT as weakly recommended against, reflecting the absence of randomized Japanese TNT data and concerns about overtreatment where baseline surgical outcomes are already favorable [60]. Since the JSCCR 2024 literature cutoff, the Japanese ENSEMBLE program has begun to address this gap: ENSEMBLE-1 and ENSEMBLE-2 established the feasibility of consolidation-based TNT [115,116], and the ENSEMBLE phase III trial is now randomizing patients to consolidation triplet CAPOXIRI versus doublet CAPOX [121]. NOM after cCR is likewise classified as weakly recommended against—with notably low consensus—reflecting higher rates of locally uncontrolled tumor growth reported in a Japanese series and the absence of validated surveillance protocols [60]. Addressing the role of TNT within this surgery-first paradigm, the ongoing phase III JCOG2207 (MARIAGE) trial is comparing short-course TNT followed by TME with selective lateral lymph node dissection against standard TME with bilateral LLND plus adjuvant chemotherapy in clinical stage III lower rectal cancer, with overall survival as the primary endpoint [130]. These divergences underscore that TNT and organ preservation strategies must be interpreted against the baseline treatment paradigm of each health system.

TNT by design delivers the full course of planned systemic chemotherapy in the preoperative setting, thereby reducing the rationale for postoperative adjuvant chemotherapy. Accordingly, neither the ESMO Clinical Practice Guideline (2025) [128] nor the ASCO Clinical Practice Guideline (2024) [113] recommends adjuvant chemotherapy after preoperative TNT as the standard of care, and no prospective trial has demonstrated additional benefit from adjuvant chemotherapy in this context [5,6,20].

### 4.3. TNT Methods: Chemotherapy Intensity and Sequencing

With TNT established as the new standard for high-risk LARC, two complementary questions emerged regarding its optimal implementation: which chemotherapy backbone to use, and when to deliver it relative to radiotherapy.

A clinically important distinction across these phase III TNT trials concerns chemotherapy intensity. RAPIDO and STELLAR employed doublet regimens (CAPOX or FOLFOX [5,114]), whereas PRODIGE 23 used triplet mFOLFIRINOX as induction [6]. An individual patient data pooled analysis of three trials—PRODIGE 23 as the triplet induction arm, with doublet arms drawn from GCR-3 and an additional phase II trial—demonstrated that triplet induction was associated with superior 5-year DFS (HR 0.67, 95% CI 0.47–0.96; *p* = 0.03) and OS (HR 0.49, 95% CI 0.31–0.78; *p* = 0.003) and a higher pCR rate, at the cost of substantially higher grade 3–4 neutropenia (17% vs. 1%; *p* < 0.0001), with no significant survival difference between doublet induction and neoadjuvant CRT alone [131]. These findings suggest that triplet-intensity induction—driven primarily by the OS benefit demonstrated in PRODIGE 23 [20]—offers superior systemic disease control, but its toxicity restricts applicability to fit patients. Randomized comparisons of doublet versus triplet backbones are now underway internationally, including the Alliance A022104/NRG-GI010 Janus Rectal Cancer Trial [120] and the Japanese ENSEMBLE phase III trial [121].

The second dimension of TNT optimization concerns the sequence of systemic chemotherapy relative to radiotherapy. Whether induction chemotherapy before CRT or consolidation chemotherapy after CRT yields superior pathological responses—and, increasingly, organ preservation rates—has emerged as a central clinical question. Against this background, CAO/ARO/AIO-12 compared induction versus consolidation FOLFOX with LC-CRT in a randomized phase II design [25]. The primary endpoint of pCR was significantly higher in the consolidation arm, while 3-year DFS and OS were equivalent, suggesting that consolidation sequencing improves tumor response without compromising systemic disease control.

OPRA directly addressed both the sequencing question and organ preservation, enrolling 324 patients with stage II–III LARC and randomizing them to induction chemotherapy followed by CRT (INCT-CRT) or CRT followed by consolidation chemotherapy (CRT-CNCT), both using FOLFOX or CAPOX and followed by response-adapted management (TME or NOM for cCR) [7]. Organ preservation (TME-free survival) was significantly higher in the consolidation arm at both 3 and 5 years, driven by a higher rate of cCR, while OS and DFS remained comparable between the arms [26]. A secondary analysis further demonstrated that clinical tumor response grade strongly predicted both organ preservation and oncological outcomes, with markedly better 3-year organ preservation and DFS for cCR than for near-complete or incomplete response (all *p* < 0.001) [132].

A pooled analysis of 628 patients from the OPRA and CAO/ARO/AIO-12 trials confirmed that watch-and-wait (W&W) does not compromise DFS, distant recurrence-free survival, local recurrence-free survival (LRFS), or OS compared with mandatory TME, supporting the oncological safety of this approach in appropriately selected patients [133]. Together, these results established the consolidation sequence as the preferred TNT approach when organ preservation is the treatment objective, as endorsed by the ASCO Clinical Practice Guideline (2024) [113] and the ESMO Clinical Practice Guideline (2025) [128].

### 4.4. Organ Preservation and Watch-and-Wait

cCR eligibility for watch-and-wait management is established through a multimodal assessment combining digital rectal examination, flexible endoscopy, and pelvic MRI [113,128,129]; biopsy is not recommended to confirm cCR, as its value in this setting is unclear [113,128]. Anterior-wall location (high salvage morbidity), cT4b disease, and clinical lateral lymph node positivity are generally regarded as relative exclusions in clinical practice.

Non-operative management as a viable oncological strategy was first systematically investigated in a prospective single-center series, in which patients with a cCR managed by observation alone achieved excellent long-term oncological outcomes [134]. This work provided an early conceptual and practical basis for NOM, subsequently termed the W&W strategy. Retrospective analyses further supported this approach, with patients achieving pCR after neoadjuvant therapy demonstrating substantially better local recurrence, OS, DFS, and distant recurrence outcomes than non-pCR patients [135].

Comprehensive real-world evidence was provided by the International Watch and Wait Database (IWWD, 2018), a registry of 880 patients with a cCR from 47 institutions across 15 countries [136]. Local regrowth occurred in approximately one-quarter of patients at 2 years; the great majority of regrowths occurred within the first 2 years, were confined to the bowel wall, and were amenable to salvage resection, with favorable 5-year overall and disease-specific survival across the cohort. Beyond oncological outcomes, the functional benefit of NOM was quantified by OnCoRe, which demonstrated that colostomy-free survival was significantly higher with W&W than with matched surgical controls [137].

However, the W&W approach carries an inherent risk. A large registry analysis pooled W&W patients with subsequent local regrowth from the IWWD and patients with a near-complete pathological response after immediate TME from the VIKINGO registry [138]. Three-year distant metastasis-free survival was significantly lower in the local regrowth group, with the greatest excess risk in patients with ypT3–4 or ypN+ disease after salvage surgery [138]. These findings underscore that patient counseling must incorporate the realistic probability that local regrowth may compromise systemic disease control, even when salvage surgery remains technically feasible. Tumor location also warrants consideration: regrowth in anterior-wall tumors, which lie close to the urogenital structures, may require more extensive salvage surgery than posterior or lateral regrowth, and this should inform shared decision-making.

Notwithstanding these risks, prospective data provide reassuring evidence in selected patients. Prospective validation came from NO-CUT, in which patients with MSS/pMMR LARC were treated with induction CAPOX followed by LC-CRT [119]. The trial achieved encouraging distant relapse-free and rectal surgery-free survival in the W&W cohort, with infrequent local regrowths—all occurring within 2 years and consistently salvageable with surgery; ctDNA negativity after TNT was independently associated with significantly better DRFS, suggesting its potential as an MRD biomarker in this population.

For patients with a good clinical response but not achieving cCR, the GRECCAR 2 trial demonstrated equivalent 5-year oncological outcomes between local excision and TME, though approximately one-third of local excision patients required completion TME due to adverse pathology [139,140].

CAO/ARO/AIO-16, a single-arm phase II study, evaluated intensified CRT followed by consolidation FOLFOX and selective W&W for cCR [117]. The trial achieved a substantial cCR rate, though local regrowth developed in a sizable proportion of W&W patients—all of whom underwent R0 salvage resection—and 3-year overall survival remained excellent. These data support the feasibility of consolidation-based W&W in European cohorts and demonstrate that TME-free patients had significantly lower low anterior resection syndrome (LARS) and Wexner scores, reflecting substantially better bowel function.

In Japan, NOMINATE was the first domestic multicenter randomized phase II trial to evaluate the feasibility of TNT and NOM in distal LARC [28], enrolling patients with distal LARC within 5 cm of the anal verge and randomizing them to consolidation chemotherapy (CRT followed by CAPOX) or a sandwich regimen (induction CAPOX plus bevacizumab, CRT, then consolidation CAPOX), with NOM offered to patients achieving cCR or near-cCR. In a companion biomarker analysis, a substantial proportion of evaluable patients achieved cCR or near-cCR and were managed with NOM, demonstrating the clinical feasibility of TNT-plus-NOM in distal Japanese rectal cancer [29]. Detailed ctDNA biomarker kinetics from this companion analysis are discussed in the dedicated ctDNA section of this review.

The oncological safety of W&W is contingent on rigorous, standardized post-treatment surveillance. Surveillance is recommended at intervals of 3–6 months for the first 2 years—the critical window during which 88.3% of local regrowths are diagnosed [136]: DRE and flexible endoscopy with biopsy provide direct luminal assessment of the scar site; pelvic MRI evaluates mesorectal and lateral nodal status; and FDG-PET/CT is reserved for equivocal or discordant cases. Serial ctDNA monitoring, evaluated in NO-CUT and NOMINATE, may help stratify distant-relapse risk and residual-disease risk; its sensitivity for local regrowth remains limited and requires prospective validation [29,119]. Most regrowths detected within this window remain amenable to curative-intent salvage resection, whereas delayed or omitted surveillance substantially compromises salvageability [136]. Harmonization and prospective validation of surveillance protocols are therefore prerequisites before W&W can be considered standard practice outside high-volume specialized centers.

### 4.5. Response-Adapted De-Escalation: PROSPECT and Beyond

The TNT trials described above enrolled predominantly high-risk LARC populations, in whom the systemic disease control benefit justifies the added toxicity of intensified multimodal therapy [5,6,114]. A parallel question concerns lower-risk locally advanced disease, for which the absolute benefit of pelvic radiotherapy—and its long-term toxicities, including radiation proctitis, anastomotic dysfunction, and sexual and urinary impairment—may not outweigh its risks. PROSPECT addressed this directly, enrolling 1194 patients with cT2N+, cT3N0, or cT3N+ rectal cancer without MRF involvement who were candidates for sphincter-sparing surgery, randomizing them to neoadjuvant FOLFOX (6 cycles) or standard long-course CRT, with CRT selectively added in the FOLFOX group only for insufficient response (less than 20% tumor regression) [8]. DFS was non-inferior in the FOLFOX group, with comparable 5-year OS and pCR rates and 5-year local recurrence below 2% in both arms. Critically, nearly nine in ten patients in the FOLFOX group ultimately avoided any CRT and its associated long-term toxicities [8].

Long-term corroboration came from the Chinese phase III FOWARC trial, in which 10-year DFS, locoregional recurrence, and OS were comparable across mFOLFOX6 alone, mFOLFOX6 plus radiotherapy, and fluorouracil plus radiotherapy [118]. These findings further support radiotherapy omission, and the ESMO Clinical Practice Guideline (2025) [128], the ASCO Clinical Practice Guideline (2024) [113], and the NCCN Guidelines (Version 2.2026) [129] all conditionally support radiotherapy omission in carefully selected MRF-negative patients.

### 4.6. Immunotherapy in MSS/pMMR Rectal Cancer and Future Directions

Selected immune checkpoint inhibitor trials in MSS/pMMR locally advanced rectal cancer (VOLTAGE-A, UNION, STELLAR II, POLAR-STAR) are summarized in Table 4B.

A critically important distinction in the immunotherapy landscape of rectal cancer lies in mismatch repair status. In contrast to MSI-H/dMMR colorectal cancer—in which immune checkpoint inhibition has emerged as the preferred systemic approach, as discussed subsequently—MSS/pMMR rectal cancer has been considered poorly immunogenic, typically exhibiting an immunologically “cold” tumor microenvironment. However, radiotherapy has been recognized as a potential immunological adjuvant that can enhance tumor immunogenicity and sensitize tumors to ICI activity through increased tumor antigen release and enhanced antigen presentation, promoting an in situ vaccine effect [141]. This rationale has driven a rapid expansion of trials evaluating immunotherapy-based neoadjuvant CRT (iNCRT) in MSS/pMMR LARC, aiming to raise complete response rates well above the 15–20% historically achieved with CRT alone [141,142].

Early single-arm trials provided proof-of-concept for this approach. VOLTAGE-A, a Japanese phase Ib/II trial, evaluated MSS/pMMR patients treated with long-course CRT followed by sequential nivolumab and demonstrated a pCR rate substantially exceeding the historical benchmark for CRT alone, with the highest pCR rates observed in patients with positive PD-L1 expression and high CD8-to-effector regulatory T-cell ratio [123]. PANDORA similarly evaluated long-course CRT followed by sequential durvalumab in predominantly pMMR patients and achieved a comparably high pCR rate with no grade 4–5 adverse events [124].

POLAR-STAR, a Chinese three-arm randomized phase II trial in all-comer patients (including both pMMR and dMMR tumors), critically evaluated the optimal sequencing of PD-1 inhibition relative to CRT [125]. Sequential PD-1 blockade after CRT (the consolidation approach) significantly improved the pCR rate compared with the CRT-only control, whereas concurrent PD-1 during CRT yielded a non-significant improvement; neither approach showed substantial differences in adverse reactions or surgical complications compared with the control.

By contrast, the NRG-GI002 trial showed that adding pembrolizumab to chemoradiotherapy after FOLFOX induction did not improve the neoadjuvant rectal (NAR) score or pCR rate, while grade 3–4 adverse events were slightly increased in the pembrolizumab arm [122].

Randomized phase III evidence came from UNION, which compared SCRT followed by CAPOX plus camrelizumab with long-course CRT followed by CAPOX in predominantly pMMR cT3–4 or N+ LARC [27]. The pCR rate was significantly higher in the immunotherapy arm than in the standard arm, with comparable surgical complication rates and similar rates of grade ≥3 treatment-related adverse events (TRAEs) across the entire treatment course, providing the most robust randomized evidence to date of a pCR benefit with iNCRT in MSS LARC.

A comprehensive meta-analysis of iNCRT approaches calculated a pooled pCR rate of 35% and MPR rate of 58%, with significantly higher rates for SCRT-based than LC-CRT-based iNCRT [142]—biologically plausible given that hypofractionated SCRT may generate greater immunogenic cell death per fraction.

Biomarker analyses across iNCRT trials have converged on a consistent immunological signature associated with a pCR, including high CD3+ stromal infiltration (*p* < 0.001) and high CD8+ stromal infiltration (*p* = 0.018), a high CD8+/eTreg ratio, low TIM-3 expression (*p* = 0.017), and PD-L1 CPS or TPS positivity [123,143], collectively suggesting that tumor immunogenicity at baseline, rather than PD-L1 expression alone, determines susceptibility to combined CRT-ICI strategies.

STELLAR II, a multicenter randomized seamless phase II/III trial, enrolled patients with MSS/pMMR LARC and compared SCRT followed by consolidation CAPOX or FOLFOX with or without concurrent sintilimab (anti-PD-1) [30]. The phase II primary endpoint of CR rate (defined as pCR or sustained cCR enabling organ preservation) was significantly higher in the immunotherapy arm, nearly doubling the CR rate and expanding eligibility for watch-and-wait [30]. Treatment completion was high in both groups; grade 3–4 TRAEs were more frequent with sintilimab, predominantly thrombocytopenia, diarrhea, and neutropenia, while grade 3–4 immune-related adverse events were uncommon [30]. The phase III primary endpoint of 3-year DFS is pending.

An important frontier beyond iNCRT is the development of radiation-free neoadjuvant immunochemotherapy (nICT) strategies that aim to preserve organ function and avoid radiation toxicities such as radiation proctitis, anastomotic fistula, and anal dysfunction. The FIRM phase II trial evaluated this approach by administering mFOLFOX6 plus serplulimab (a PD-1 inhibitor) in MSS/pMMR LARC and achieved pCR and MPR rates substantially exceeding those reported for neoadjuvant chemotherapy alone and the historical CRT benchmark, with comparable activity to CRT-based iNCRT approaches [126]. This approach is particularly applicable to high rectal tumors (greater than 10 cm from the anal verge) not amenable to standard pelvic CRT, in which the FIRM trial achieved especially high pCR and MPR rates. Despite frequent grade 3 TRAEs, no grade 4–5 TRAEs or anastomotic fistula were reported, and the regimen was deemed manageable; the randomized FIRM-02 trial is underway to validate this approach.

**Figure 3:**
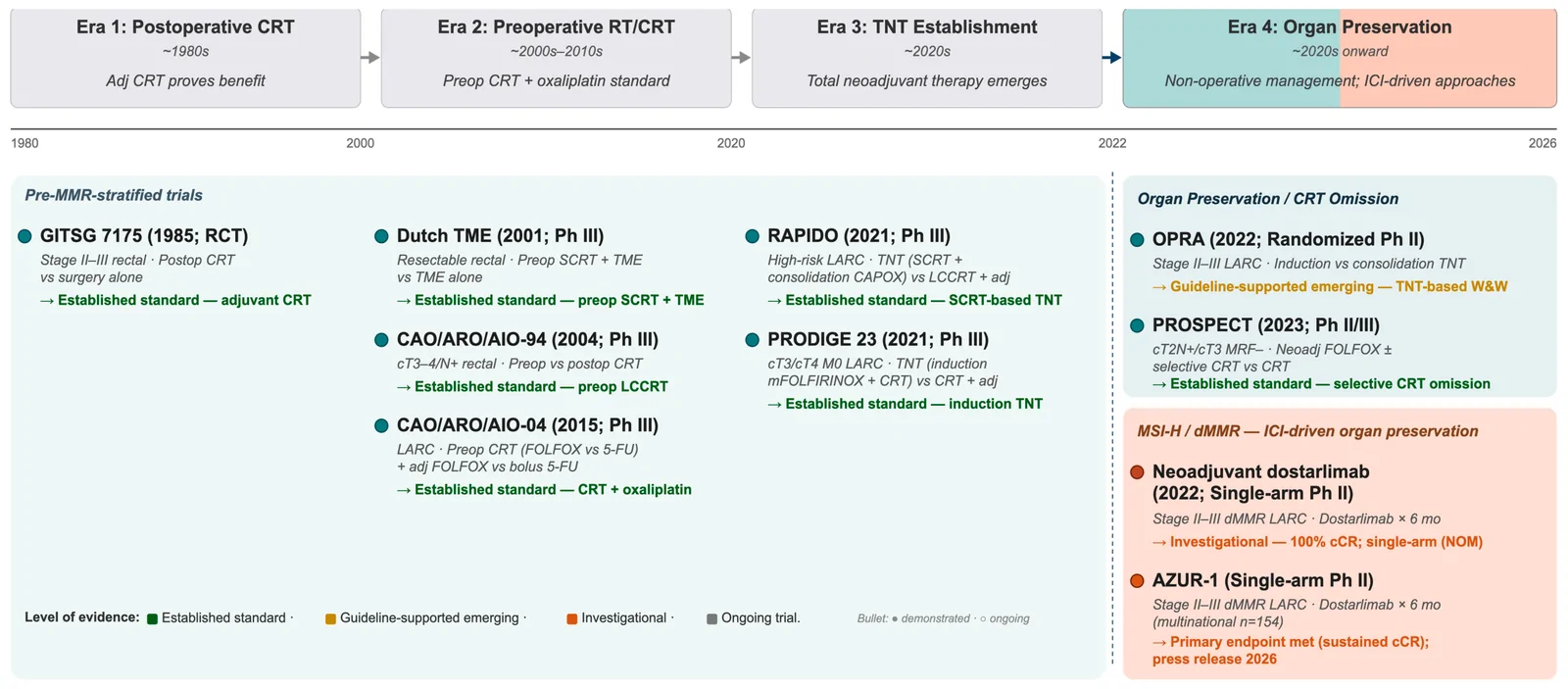
Paradigm shifts in rectal cancer perioperative therapy, from postoperative chemoradiotherapy through TNT to organ preservation and biology-stratified ICI.

Synthesizing this evidence, a consistent pattern is emerging in MSS/pMMR LARC: SCRT followed by consolidation chemotherapy and sequential PD-1 blockade is associated with the highest reported cCR and pCR rates in cross-trial comparison—as shown by UNION (camrelizumab) [27] and STELLAR II (sintilimab) [30]—substantially exceeding both the 15–20% historically achieved with CRT alone [141,142] and the approximately 28% reported with TNT chemotherapy [5,6]. These approaches remain investigational, however, and whether the pathological response gains translate into improved disease-free and overall survival is the critical unanswered question, pending ongoing phase III endpoints (STELLAR II, UNION long-term follow-up, POLAR-STAR). The optimal sequencing of radiotherapy, chemotherapy, and immunotherapy, and the potential role of dual checkpoint blockade, define the frontier for future research. Thus, whereas ICI acts as a response-enhancing adjunct under active investigation in pMMR rectal cancer, in MSI-H/dMMR rectal cancer (discussed in the following section) it emerges as a stand-alone strategy for organ preservation. Paradigm shifts in rectal cancer perioperative therapy, from postoperative CRT through TNT to organ preservation and biology-stratified ICI, are illustrated in Figure 3.

## 5. MSI-H/dMMR Rectal Cancer: Immunotherapy-Driven Organ Preservation

In MSI-H/dMMR rectal cancer—a rare subtype with disproportionate sensitivity to immune checkpoint inhibition—the pivotal paradigm shift was the demonstration that single-agent neoadjuvant dostarlimab achieved a complete clinical response in essentially all treated patients within small, molecularly selected phase II cohorts [22,31], raising the possibility that immunotherapy alone might serve as definitive treatment and obviate both pelvic surgery and radiation. This evidence, while remarkable, remains non-randomized and is currently limited by modest sample sizes and short follow-up. Building on this proof-of-concept, current frontiers include international multicenter confirmation (AZUR-1), evaluation of alternative regimens such as ICI plus short-course radiotherapy (EA2201) and ultra-short dual checkpoint blockade (RESET-R), and the identification of biomarkers to refine patient selection.

### 5.1. Epidemiology and Molecular Rationale of MSI-H/dMMR Rectal Cancer

dMMR or MSI-H rectal cancer is a rare molecular subset, accounting for approximately 5–10% of all rectal adenocarcinomas [31]—substantially lower than in non-metastatic colon cancer [42]. Unlike sporadic dMMR colon cancer, which predominantly arises through *MLH1* promoter hypermethylation and *BRAF* V600E mutation, dMMR rectal cancer is more commonly associated with Lynch syndrome germline MMR gene mutations, with implications for familial risk counseling [144]. In a prospective cohort of 482 unselected consecutive CRC patients, dMMR was identified in only 3.1% of rectal cancers versus 14.6% of colon cancers; notably, all dMMR rectal cancers in this cohort showed *MSH2*/*MSH6* deficiency without *MLH1*/*PMS2* loss, confirming the predominance of the Lynch syndrome pathway over the sporadic hypermethylation pathway in this site [144]. Consistent with this Lynch syndrome predominance, *BRAF* V600E—a hallmark of the sporadic dMMR colon cancer pathway—was absent in the patients tested in both cohorts [31,144], and its near-universal absence in dMMR LARC precludes formal subgroup analysis of ICI response by *BRAF* status; a single case of *BRAF* V600E-mutant dMMR rectal cancer progressing on dostarlimab has been described, although co-occurring mutations preclude attribution to *BRAF* status alone [145]. Whether differences in the specific germline MMR gene defect (*MSH2*, *MSH6*, *MLH1*, or *PMS2*) modulate depth of response to PD-1 blockade in the LARC setting has not been formally evaluated in prospective studies.

The molecular basis of ICI sensitivity in dMMR tumors is detailed in Section 3; the same hallmark features—high neoantigen burden, elevated tumor mutational burden, and dense intratumoral T-cell infiltration—establish MSI-H/dMMR CRC as among the most ICI-responsive malignancies [85,86,146] and provide the rationale for investigating whether this sensitivity can achieve responses deep enough to obviate both surgery and radiation in LARC [22,31].

The clinical imperative for this question is heightened by the unique stakes of rectal cancer treatment. TME combined with neoadjuvant CRT constitutes the historical standard of care for LARC [113,128,129], but carries a substantial burden of long-term morbidity—permanent colostomy, LARS, sexual dysfunction, urinary impairment, and infertility—that is of particular concern in younger patients. These considerations make organ preservation, and specifically the elimination of both pelvic surgery and radiation, a compelling goal in dMMR LARC and provide the imperative for the immunotherapy-only strategies described below [22,31].

### 5.2. From CRT Plus ICI to Immunotherapy Alone as a Definitive-Intent Organ Preservation Strategy for dMMR LARC

VOLTAGE-A was the first trial to evaluate CRT plus PD-1 blockade in both MSS and MSI-H LARC [11,123]. In this Japanese open-label single-arm phase Ib/II study, the primary MSS cohort received preoperative CRT followed by sequential nivolumab and TME, and the exploratory MSI-H cohort the same regimen with mandatory TME; the MSI-H cohort achieved a high pCR rate, and 3-year follow-up confirmed excellent RFS and OS without recurrence [11,123]. A meta-analysis of six randomized trials in LARC regardless of MMR status found that adding PD-1 inhibitors to neoadjuvant CRT significantly improved pCR rates (OR 2.10, 95% CI 1.32–3.32; *p* = 0.001) without increasing serious adverse events; notably, the benefit was confined to pMMR patients, consistent with a ceiling effect in tumors whose baseline immunogenicity is already high—a finding that motivated evaluating whether ICI monotherapy alone could suffice in dMMR LARC [147].

These observations prompted the investigation of whether immunotherapy alone, without CRT, surgery, or chemotherapy, could achieve sufficient tumor eradication in dMMR LARC to obviate multimodal treatment. In a phase II single-group study, patients with dMMR stage II or III rectal adenocarcinoma received single-agent dostarlimab for 6 months; those achieving cCR proceeded without CRT or surgery, while those without cCR received standard CRT followed by TME [31]. All 12 evaluable patients (100%) achieved cCR in the initial report [31], and an updated analysis extending to 49 patients confirmed 100% cCR with a 2-year RFS of 96% at a median follow-up of 30.2 months [22]. Grade 3–4 adverse events occurred in five patients with no treatment-related deaths, and ctDNA clearance within a median of 1.4 months showed high concordance with biopsy-confirmed response (kappa 0.76) [22]. These results provided the first prospective evidence that dMMR LARC can achieve sustained clinical complete response with immunotherapy alone in a molecularly selected population [22,31]. Subsequent real-world series from the United States and Europe have provided independent validation across different PD-1 inhibitors and patient populations [148,149].

Following publication of the initial phase II findings, early real-world adoption of neoadjuvant anti-PD-1 therapy at four US academic centers was captured in a retrospective multisite series of dMMR LARC patients (predominantly pembrolizumab or dostarlimab) [148]. The majority of patients achieved an objective response, with approximately half achieving cCR; among the non-cCR patients, salvage CRT and surgery yielded pCR in a substantial proportion, and 2-year DFS was favorable at limited follow-up [148]. The lower cCR rate in this retrospective series compared with the phase II trial [31] likely reflects variable treatment duration, patient heterogeneity, and retrospective ascertainment [148].

A European counterpart to the US real-world experience was provided by the STAR(t)-IT-REDUCE study, an observational retrospective-cohort multicentric study conducted across 12 Italian centers [149]. MSI-H/dMMR LARC patients (predominantly dostarlimab or pembrolizumab, with a minority receiving nivolumab plus ipilimumab) were eligible, with most being treatment-naïve. The majority of patients achieved cCR on ITT analysis, and the cCR rate among patients who completed the protocol-defined 6-month treatment was likewise high. At limited follow-up, no local relapses occurred and all patients were alive, with only one patient developing unconfirmed lung metastases. Grade 3 adverse events were uncommon and no grade 4 events occurred [149]. The higher cCR rate in this series compared with the US real-world cohort likely reflects longer median treatment duration in the Italian cohort. The characteristics of non-responding patients in real-world series—including mucinous histology, shorter treatment duration, and the potential contribution of MMR intratumoral heterogeneity (whereby focal MMR-proficient subclones within an overall dMMR tumor may escape immunosurveillance and drive primary resistance)—represent an important area for prospective investigation [145].

A distinct approach to maximizing response depth is the use of dual checkpoint blockade. In a multicenter single-arm phase II study conducted in Denmark (RESET-R), patients with dMMR rectal cancer received only 1 or 2 cycles of neoadjuvant nivolumab plus ipilimumab [32]. All patients achieved cCR and proceeded to a W&W strategy; no recurrences were observed at limited follow-up, and grade 3 adverse events were uncommon with no deaths. These interim results suggest that ultra-short dual ICI may achieve responses comparable to longer single-agent regimens, though confirmation with longer follow-up and larger cohorts is required [32].

Taken together, these studies establish a coherent biological principle: dMMR rectal cancer is profoundly sensitive to PD-1 blockade across multiple agents, institutions, and patient populations [31,148,149]. Treatment duration appears to be a critical determinant of cCR probability, with 6 months of single-agent dostarlimab (9 cycles) representing the most mature evidence base [22,31].

Dual checkpoint blockade with nivolumab plus ipilimumab, administered in as few as 1 or 2 cycles in the RESET-R study, achieved cCR in all patients of a small Danish cohort, with the majority achieving cCR after only one cycle [32]. The authors of RESET-R noted that the use of dual rather than single-agent blockade may partly explain the shorter time to cCR compared with the 6-month dostarlimab monotherapy regimen—raising the hypothesis that blockade intensification may compensate for reduced duration, though this remains unproven in a comparative trial [32]. As demonstrated in dMMR colon cancer (NEOSHOT), dual checkpoint blockade can significantly improve the pathological complete response rate compared with PD-1 monotherapy [90]; whether this advantage translates to superior cCR rates in dMMR LARC remains to be tested. Direct randomized comparison of single-agent and dual-agent regimens in dMMR LARC is currently lacking, and the relative merits of each approach—including long-term organ preservation rates, durability, and toxicity—require prospective evaluation.

### 5.3. Implementation of Organ Preservation After Immunotherapy

The cCR rates achieved with immunotherapy alone have substantially changed the management landscape for dMMR LARC [22,31], but the practical implementation of NOM requires careful attention to challenges that differ fundamentally from the W&W experience following CRT in pMMR patients. Accurate multimodal assessment of cCR is the cornerstone of safe NOM implementation [22,31]. The dostarlimab phase II protocol defines cCR by a rigorous multimodal standard that requires concordant complete response on all of the following: digital and endoscopic rectal examination with biopsy, rectal MRI (with no restricted diffusion on T2-weighted sequences), and FDG-PET/CT [22,31]. A retrospective French cohort study analyzed MRI response patterns in patients with dMMR rectal cancer who had all achieved confirmed complete response after anti-PD-1 monotherapy [150]. MRI response patterns after ICI differed substantially from those observed after CRT, with heterogeneous appearances including fibrotic responses, residual intermediate T2 signal, split scar signs, and complete lesion disappearance—making standard CRT-based MRI response grading criteria (mrTRG) not directly applicable to this treatment context [150]. All mucinous tumors retained T2-hyperintense residues despite confirmed complete response, illustrating the particular risk of imaging-based misclassification in this histological subtype, and patients imaged early sometimes showed delayed fibrotic responses on subsequent re-imaging, suggesting that prolonged imaging surveillance may reduce false positive classification of residual disease [150].

These single-center observations are supported by a systematic meta-analysis of 12 studies [151]. In the rectal cancer subgroup, the radiological–pathological discordance rate was substantially lower than in colon cancer, reflecting the availability of multimodal assessment including DRE, flexible endoscopy, and pelvic MRI, and nearly all rectal cancer patients achieving cCR had confirmed pCR or no recurrence within 6 months [151]. These data confirm that endoscopic biopsy remains indispensable for cCR confirmation, and that imaging abnormalities after ICI must not be used as the sole basis for a decision to proceed to salvage surgery [151].

Structured surveillance after NOM is essential, though optimal protocols remain formally undefined. In the dostarlimab phase II protocol [31], surveillance is performed every 4 months, incorporating rectal MRI, endoscopy with biopsy, and FDG-PET/CT [31]. The potential role of serial ctDNA monitoring as an early molecular indicator of local regrowth or distant recurrence during NOM surveillance—complementing conventional endoscopic and radiological assessment—is an active area of investigation; available evidence in this context and the broader framework of ctDNA-guided surveillance are addressed in a dedicated section of this review.

### 5.4. Remaining Challenges and Future Directions

The accumulating evidence has been incorporated into major international guidelines. The ESMO Clinical Practice Guideline (2025) recommends immunotherapy for patients with locally advanced dMMR or MSI-H rectal cancer, and endorses a watch-and-wait strategy for those achieving cCR [128]. The ASCO Clinical Practice Guideline (2024) similarly recommends immunotherapy for MSI-H or dMMR rectal cancer [113], and the NCCN Guidelines (Version 2.2026) include checkpoint inhibitor immunotherapy as a recommended treatment option for dMMR rectal cancer [129]. However, formal regulatory approval of dostarlimab specifically for locally advanced dMMR rectal cancer has not yet been granted by the FDA or EMA; the FDA granted Breakthrough Therapy Designation in December 2024, acknowledging the strength of the available evidence [152], but full approval for this specific indication is not yet established. Accordingly, although immunotherapy for dMMR rectal cancer is guideline-recommended, the immunotherapy-only non-operative decision itself rests on single-arm, non-randomized evidence and awaits mature, peer-reviewed results from the multicenter AZUR-1 trial [153]—whose primary endpoint was recently reported to be met by press release [154]—and, ultimately, randomized confirmation.

Despite guideline endorsement, the question of whether immunotherapy should be offered to all patients with dMMR LARC warrants consideration. Absolute contraindications to immune checkpoint inhibition—including active autoimmune disease requiring systemic immunosuppression and prior solid organ transplantation—preclude ICI in a subset of patients, for whom standard CRT-based management remains the appropriate treatment [113]. Additionally, patients who decline NOM and prefer definitive surgical resection, or those with early-stage (stage I) disease in whom the risk-benefit calculation for immunotherapy differs from locally advanced disease, represent populations in whom the universal application of ICI is debated [128]. Patient preference for immediate certainty of surgical cure versus the uncertainty of NOM surveillance also constitutes a legitimate clinical consideration that guidelines acknowledge but cannot mandate [113,129].

Several fundamental uncertainties temper these results. No randomized trial has yet compared immunotherapy-only non-operative management with standard chemoradiotherapy and TME, and even the principal confirmatory study, AZUR-1, is a single-arm phase II trial; the long-term durability of organ preservation and the efficacy of salvage therapy after late local regrowth in this dMMR population therefore remain incompletely defined. Long-term durability data are limited, with a median follow-up of 30.2 months in the rectal cancer cohort [22], and primary resistance to PD-1 blockade does occur: a small case series described three patients with dMMR LARC who experienced disease progression on dostarlimab, with mucinous histology and transcriptomic signatures implicated as potential contributors to intrinsic resistance [145]. These observations underscore the need for biomarkers capable of predicting non-response before treatment initiation [145].

Several large prospective trials are now underway to provide international confirmation and to address the key limitations of the single-center dataset [22,31]. AZUR-1 (NCT05723562) is a single-arm phase II study of upfront dostarlimab in MSI-H/dMMR LARC conducted across 10 countries, in which 154 participants received treatment [153,154]. Patients receive dostarlimab monotherapy (500 mg intravenously every 3 weeks for 6 months); those achieving cCR proceed to NOM, with clinical complete response at 24 and 36 months and 3-year event-free survival as key secondary endpoints, and organ preservation at 3 years among the additional secondary endpoints [153]. In July 2026, the sponsor announced by press release that AZUR-1 had met its primary endpoint of a sustained clinical complete response at 12 months, although the specific response rate has not yet been disclosed and mature, peer-reviewed results await presentation at a forthcoming congress [154]. By providing international multicenter data with planned 5-year follow-up, AZUR-1 is intended to address long-term durability—the principal evidence gap identified in the initial reports—although randomized confirmation against standard chemoradiotherapy and TME remains lacking [22,31].

EA2201 (NCT04751370), conducted by ECOG-ACRIN, is a phase II single-arm study evaluating neoadjuvant nivolumab plus ipilimumab combined with short-course radiation (SCRT) in patients with MSI-H/dMMR stage II–III locally advanced rectal cancer, with pathological complete response at total mesorectal excision as the primary endpoint [155]. APRAM (NCT05669092) is a randomized phase III study evaluating MMR status–stratified neoadjuvant treatment for low rectal adenocarcinoma (within 5 cm from the anal verge), with ICI alone versus short-course radiotherapy (SCRT) followed by ICI in the MSI-H/dMMR stratum [156], providing data on whether radiation enhances dMMR tumor responses to PD-1 blockade in the distal rectal setting.

Dual checkpoint blockade and molecular subgroup stratification represent parallel avenues for future investigation. Nivolumab plus ipilimumab—as demonstrated in the metastatic setting by CheckMate 8HW [86,146]—represents a rational escalation strategy for dMMR LARC patients who fail to achieve complete response with single-agent PD-1 blockade that has not yet been prospectively evaluated in the non-metastatic setting. Within the dMMR population, additional biomarkers—including specific MMR gene defects (Lynch syndrome MSH2 vs. sporadic *MLH1* methylation) [144], pretreatment CD8+ tumor-infiltrating lymphocyte density, and early ctDNA kinetics [22,92]—may further stratify response probability and inform individualized treatment duration decisions.

Immunotherapy trials in MSI-H/dMMR rectal cancer are summarized in Table 5.

**Table 5:** Pivotal Trials of Perioperative Therapy in MSI-H/dMMR Rectal Cancer.

Trial (Year)	N	Setting	Regimen	cCR/pCR Rate	DFS or RFS (Follow-Up)	Reference	Level of Evidence ‡
Cercek dostarlimab (2022; Single-arm Ph II)	12 evaluable	Neoadjuvant, stage II–III	Dostarlimab monotherapy × 9 (6 mo)	cCR 100% (12/12)	No recurrence at initial report	[31]	Guideline-supported emerging (single-arm)
— Cercek update (2025; Single-arm Ph II)	49	Neoadjuvant, stage II–III	Same	cCR 100%	2 yr RFS 96% at median 30.2 mo	[22]	Guideline-supported emerging (single-arm)
VOLTAGE-A MSI-H cohort (2022; Ph Ib/II)	5	Neoadjuvant, LARC	LC-CRT (50.4 Gy + capecitabine) → nivolumab × 5 → TME	pCR 60%	—	[11,123]	Investigational (small cohort)
— VOLTAGE-A 3 yr (2024; Ph Ib/II)	5	Neoadjuvant, LARC	Same	pCR 60%	3 yr RFS and OS both 100% (median follow-up 56.4 mo)	[11]	Investigational (small cohort)
STAR(t)-IT-REDUCE (2024; real-world cohort)	17	Neoadjuvant, LARC (real-world)	Dostarlimab 59%, pembrolizumab 35%, nivolumab + ipilimumab 6%	cCR 94.1% (16/17, ITT); 90.9% protocol-complete	No local relapse; all alive at median 9.5 mo	[149]	Investigational (real-world)
NEOSHOT (2025; Randomized Ph Ib) ¶	—	Neoadjuvant, dMMR colon (not rectal)	IBI310 (anti-CTLA-4) + sintilimab vs. sintilimab alone	pCR 78.4% vs. 46.7%; *p* = 0.0015	— (dMMR colon, not rectal)	[90]	Investigational (context; dMMR colon)
US real-world (Emiloju 2026 [148]; real-world)	21	Neoadjuvant, LARC (real-world)	Anti-PD-1 (various: pembrolizumab/dostarlimab/nivolumab; one ipi + nivo)	ORR 85.7%; cCR 52.4%	2 yr DFS 87% at median 19 mo	[148]	Investigational (real-world)
RESET-R (2026; Ph II)	16	Neoadjuvant, stage I–III	Nivolumab + ipilimumab (1–2 cycles)	cCR 100% (16/16)	No recurrence at median 16 mo	[32]	Investigational (single-arm)
**— Ongoing trials —**							
APRAM (NCT05669092; Ph III)	TBD	Neoadjuvant, low rectal (≤5 cm from anal verge)	ICI alone vs. SCRT → ICI in MSI-H/dMMR stratum	TBD	Ongoing	[156]	Ongoing
AZUR-1 (NCT05723562; Single-arm Ph II)	154 treated	Neoadjuvant, LARC	Multinational; dostarlimab monotherapy ×6 mo, then NOM for cCR	cCR by independent central review at 12 mo	Primary endpoint met (sustained cCR); rate not yet disclosed (press release 2026); cCR at 24 and 36 mo and 3 yr EFS as key secondary endpoints; organ preservation at 3 yr among additional secondary endpoints	[153,154]	Ongoing
EA2201 (NCT04751370; Single-arm Ph II)	31 (planned)	Neoadjuvant, stage II–III	Nivolumab + ipilimumab + SCRT (ECOG-ACRIN/NCI)	pCR at TME	Active, not recruiting; primary completion 31 December 2026	[155]	Ongoing

*Note:* ¶ NEOSHOT enrolled dMMR colon cancer (not rectal); included as the principal randomized reference establishing dual checkpoint blockade superiority in dMMR CRC, cited as biological context for dual-ICI strategies in dMMR LARC. *Abbreviations:* cCR, clinical complete response; CTLA-4, cytotoxic T-lymphocyte-associated protein 4; DFS, disease-free survival; dMMR, deficient mismatch repair; ICI, immune checkpoint inhibitor; ITT, intention-to-treat; LARC, locally advanced rectal cancer; LC-CRT, long-course chemoradiotherapy; MMR, mismatch repair; MSI-H, microsatellite instability-high; NOM, non-operative management; ORR, objective response rate; OS, overall survival; PD-1, programmed cell death protein 1; pCR, pathological complete response; RFS, recurrence-free survival; SCRT, short-course radiotherapy; TBD, to be determined; TME, total mesorectal excision. ‡ Evidence-level classification as defined in the footnote to Table 1.

## 6. ctDNA-Based Minimal Residual Disease (MRD) Assessment

ctDNA has emerged as a cross-cutting biomarker that spans the molecular subtypes and primary sites discussed in the preceding sections, providing a unifying framework for adaptive perioperative management. Four pivotal trials define the current evidence base: GALAXY established the prognostic power of postoperative ctDNA at large scale [35,36], DYNAMIC provided the first randomized proof that ctDNA-guided de-escalation is non-inferior to standard adjuvant therapy in stage II colon cancer [33,34], ALTAIR, whose pre-specified primary analysis did not reach significance, provided an early activity signal for trifluridine/tipiracil in ctDNA-persistent disease [37], and an interim analysis of the phase III FIND trial provided the first randomized signal that ctDNA-based surveillance increases the proportion of recurrences treated with curative intent, pending mature results [39]. Current frontiers include prospective validation of ctDNA-guided escalation and integration with multimodal risk frameworks.

### 6.1. Biological and Technical Determinants of ctDNA Detection

ctDNA refers to tumor-derived DNA fragments carrying somatic alterations that are shed into the bloodstream and detectable in plasma, enabling non-invasive molecular monitoring of residual disease after curative-intent surgery [157]. Two broad detection strategies underpin the trials discussed in this section. Tumor-informed (TI) assays use prior whole-exome or targeted sequencing of the primary tumor to design patient-specific panels—exemplified by Signatera and RaDaR—achieving high analytical sensitivity through personalized variant tracking, at the cost of requiring tumor tissue and a design turnaround of several weeks [157]. Tumor-agnostic (plasma-only) assays, such as Guardant Reveal (combining somatic mutation and methylation signals) and methylation-based multiplex PCR platforms, circumvent the need for tumor tissue by targeting recurrently altered genomic or epigenomic features validated in large prospective CRC cohorts [158,159]. The distinction between these approaches is clinically relevant because the major perioperative trials differ in their assay choice: DYNAMIC and CIRCULATE-Japan employ TI assays, whereas PEGASUS and FIND use tumor-agnostic platforms.

Two technical considerations are germane to perioperative applications. For mutation-based assays, somatic mutations arising in hematopoietic cells (clonal hematopoiesis of indeterminate potential, CHIP) can generate false positive cell-free DNA (cfDNA) signals that mimic ctDNA; TI assays mitigate this through matched white blood cell sequencing to filter germline and CHIP variants from the patient-specific panel, whereas mutation-based tumor-agnostic platforms require algorithmic filtering at each sampling timepoint [157]. Methylation-based assays are largely unaffected by CHIP. Regardless of assay type, surgical trauma transiently elevates cfDNA in the immediate postoperative period; standardized sampling at week 4 and week 7 post surgery—as employed in the DYNAMIC trial—is therefore required to allow for the clearance of surgery-derived signals before MRD assessment [33]. A systematic review and diagnostic accuracy meta-analysis directly comparing TI and TA assays in resected CRC demonstrated that TI assays achieve significantly higher sensitivity than TA assays in serial monitoring settings (0.88 vs. 0.59; *p* = 0.001), with no significant difference in false positive rates; this sensitivity advantage was not observed in single-timepoint (landmark) analyses [160].

Emerging next-generation TI platforms further differ in their underlying sequencing strategy. Whole-genome sequencing (WGS)-based MRD assays—exemplified by the SCRUM-Japan MONSTAR-SCREEN-3 platform—interrogate large numbers of tumor-specific variants across the genome and have been preliminarily reported to achieve detection sensitivity at the parts-per-million range (mutant allele frequencies of approximately 10^−6^), below the analytical floor of conventional multiplex PCR–next-generation sequencing (NGS) TI panels [161]. In parallel, integrated whole-exome plus whole-transcriptome sequencing platforms leverage matched tumor genomic and transcriptomic profiling for tissue-informed monitoring [157,158]. Because false positive rates were comparable between the two approaches while sensitivity favored TI assays [160], false negative results may be more likely with TA assays in longitudinal de-escalation contexts—a consideration bearing on the generalizability of findings across trials using different platforms.

Beyond platform choice, tumor-intrinsic biological factors further modify ctDNA shedding probability and detection rates across CRC subtypes [35,162]. dMMR tumors present a particular challenge for ctDNA-based MRD monitoring. Despite their high immunogenicity and responsiveness to immune checkpoint inhibitors, dMMR tumors are characteristically associated with lower levels of ctDNA shedding compared with pMMR tumors—a pattern hypothesized to reflect immune-mediated suppression of micrometastatic disease. In GALAXY, the MRD window positivity rate among MSI-H patients was only 2.8%, against an overall rate of 15.9%, confirming markedly reduced ctDNA shedding in MSI-H tumors [35,36]. Reflecting their favorable underlying prognosis, MSI-H patients had better DFS than MSS patients irrespective of ctDNA status [35,36]. Because dMMR tumors shed substantially less ctDNA, a tumor-informed assay would be expected to have correspondingly lower clinical sensitivity for residual disease when it is present in this subtype. While this limitation is partially offset by the more favorable baseline recurrence risk of MSI-H disease [36], it warrants caution before extrapolating ctDNA-guided de-escalation strategies—validated primarily in the MSS/pMMR population—to MSI-H/dMMR patients [35,36].

**Figure 4:**
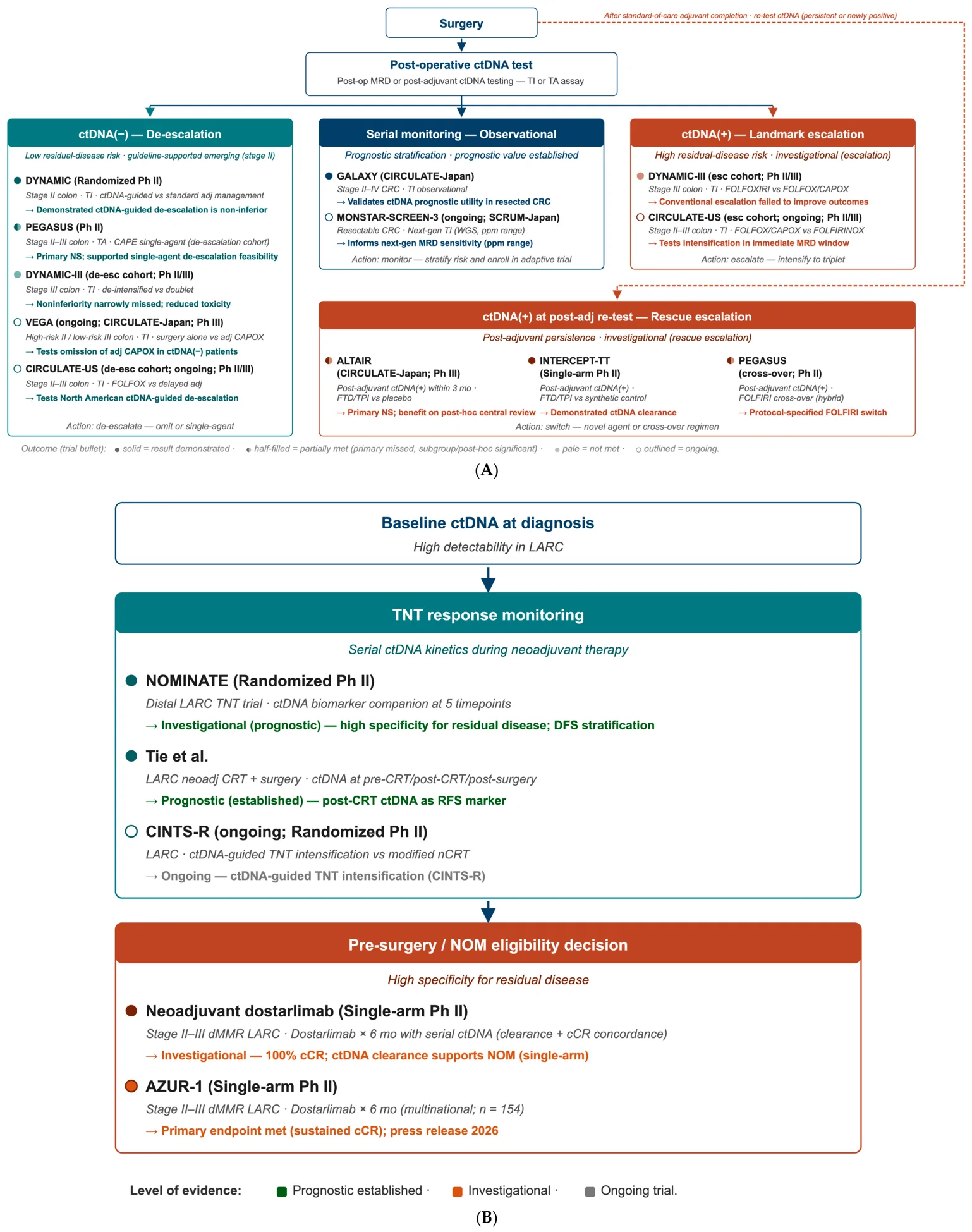
(**A**) ctDNA-guided postoperative MRD framework in resected colorectal cancer: parallel tracks of treatment de-escalation in ctDNA-negative patients, observational serial monitoring, and therapeutic escalation in ctDNA-positive patients. NS, not significant. (**B**) ctDNA dynamics during neoadjuvant treatment and watch-and-wait surveillance in locally advanced rectal cancer. Trials shown: DYNAMIC [33]; PEGASUS [41]; DYNAMIC-III [38,163]; VEGA [164]; CIRCULATE-US [165]; GALAXY (CIRCULATE-Japan) [35,36]; MONSTAR-SCREEN-3 [161]; ALTAIR [37]; INTERCEPT-TT [40]; NOMINATE [29]; Tie et al. [166]; CINTS-R [167,168]; neoadjuvant dostarlimab [31]; AZUR-1 [153,154].

Primary tumor sidedness similarly influences ctDNA detection rates. In GALAXY, right-sided primaries were proportionally under-represented among ctDNA-positive patients, whereas left-sided colon and rectal tumors were over-represented (*p* = 0.001) [35]. This distribution reflects in part the higher prevalence of the MSI-H/dMMR lower-shedding phenotype in right-sided cancers; the biological determinants beyond the MSI effect remain under investigation. The relationship between ctDNA, sidedness, and molecular subtypes (*BRAF* V600E, *RAS* mutation) is being characterized within platforms such as GALAXY [36], with the goal of developing subgroup-specific ctDNA thresholds and interpretation frameworks. The ctDNA-guided perioperative decision framework comprises two complementary paradigms: the postoperative MRD framework in resected colorectal cancer—comprising three parallel tracks of treatment de-escalation in ctDNA-negative patients, observational serial monitoring (e.g., GALAXY, MONSTAR-SCREEN-3) providing risk stratification and lead time over imaging, and therapeutic escalation in ctDNA-positive patients—is illustrated in Figure 4A, while ctDNA dynamics during neoadjuvant treatment and watch-and-wait surveillance in locally advanced rectal cancer are summarized in Figure 4B.

### 6.2. Prognostic Value of Postoperative ctDNA in Colorectal Cancer

The prognostic value of postoperative ctDNA in resected CRC was established in prospective studies demonstrating that ctDNA positivity identifies patients at extremely high recurrence risk and—critically for perioperative management—those who derive benefit from adjuvant chemotherapy. In stage II colon cancer, ctDNA positivity in patients not receiving adjuvant chemotherapy was associated with a markedly elevated recurrence risk, and persistent ctDNA after adjuvant treatment completion similarly predicted inferior recurrence-free survival [169]. These findings were extended to locally advanced rectal cancer, where postoperative ctDNA positivity predicted recurrence and remained an independent predictor after adjustment for clinicopathological factors [166].

The largest prospective validation dataset came from the GALAXY study, the multicenter observational registry arm of the Japanese CIRCULATE-Japan platform. GALAXY prospectively enrolled patients with stage II–IV colorectal adenocarcinoma undergoing R0 curative-intent resection across 92 institutions nationwide, with serial tumor-informed (Signatera) testing from week 4 after surgery [35]. In the updated analysis of 2240 evaluable patients, postsurgical ctDNA positivity was the single most significant prognostic factor on multivariate analysis, conferring markedly inferior DFS and OS, and adjuvant chemotherapy was associated with significant benefit in ctDNA-positive but not ctDNA-negative patients [36]. Sustained ctDNA clearance during adjuvant chemotherapy was associated with markedly superior DFS compared with transient clearance, and spontaneous clearance without recurrence occurred in only 1.9% of ctDNA-positive patients, consistent with the high positive predictive value of the assay [36].

A dedicated rectal cancer subgroup analysis of stage II–III patients undergoing upfront surgery identified ctDNA positivity in approximately 14%, with markedly shorter DFS in ctDNA-positive patients; adjuvant chemotherapy was associated with significant benefit in ctDNA-positive but not in ctDNA-negative patients, and longitudinal dynamics further stratified risk, with conversion to positivity by 6 months and persistent positivity conferring substantially worse outcomes than serial negativity [170].

ctDNA detects recurrence ahead of conventional radiological surveillance in most series, with a median lead time of 7.3 months (IQR 3.3–12.5) reported in the UK TRACC Part B study and syntheses describing windows of up to 9 months [158,171]. While the robust prognostic value of postoperative ctDNA is well-established, demonstrating its clinical utility—specifically, whether altering treatment based on ctDNA status improves patient outcomes—represents the next critical hurdle. Consequently, the paradigm is shifting from observational prognostication to ctDNA-guided interventional trials.

### 6.3. ctDNA-Guided Adjuvant Therapy: De-Escalation and Escalation Strategies

ctDNA-guided adjuvant therapy encompasses two contrasting strategies anchored to opposite ends of the molecular risk spectrum: de-escalation in ctDNA-negative patients, who carry a low residual recurrence risk, and escalation in ctDNA-positive patients, in whom molecular residual disease portends a markedly elevated risk of relapse. This subsection examines both strategies in turn, beginning with the de-escalation evidence base—where prospective randomized data have matured fastest—before turning to the more biologically challenging question of treatment escalation.

The first interventional question posed for ctDNA-guided management was whether ctDNA-negative patients—who carry a low residual recurrence risk—can safely avoid or de-intensify adjuvant chemotherapy. DYNAMIC was the first randomized controlled trial to test whether ctDNA-guided decision-making could safely reduce adjuvant chemotherapy use in stage II colon cancer, randomizing 455 patients 2:1 to ctDNA-guided or standard clinicopathological management [33]. In the ctDNA-guided arm, blood collected at 4 and 7 weeks after surgery was analyzed concurrently; patients positive at either timepoint received chemotherapy, while those negative at both received none. The primary endpoint of 2-year RFS non-inferiority was met (non-inferiority margin −8.5 percentage points), with lower adjuvant chemotherapy use in the ctDNA-guided group [33].

Mature 5-year outcomes confirmed the durability of these findings, with virtually identical RFS and OS between the arms [34]. Post hoc analyses further showed that ctDNA burden stratified risk among ctDNA-positive patients and that ctDNA clearance at the end of adjuvant therapy predicted a markedly higher 5-year recurrence-free probability than persistent ctDNA (*p* < 0.001) [34]. These results should be interpreted alongside critical commentary noting that the non-inferiority margin of 8.5 percentage points may be clinically too broad, and that real-world implementation could paradoxically increase chemotherapy use if ctDNA testing is applied additively rather than as a replacement for clinicopathological risk stratification [172].

Complementary validation of ctDNA-guided de-escalation using a tissue-free (plasma-only) assay was provided by PEGASUS (ESMO 2025), a multicenter phase II study employing the Guardant Reveal assay in stage III and high-risk stage II colon cancer [41]. Among ctDNA-negative patients assigned to de-escalated capecitabine monotherapy, the 2-year non-evidence-of-disease rate exceeded the prespecified threshold in the point estimate, although formal significance was not met, in part because of under-enrollment of that arm; ctDNA-positive patients relapsed substantially more often, reinforcing the adverse prognostic significance of ctDNA positivity. The trial also incorporated a pre-specified sequential strategy in which ctDNA-positive patients received 3 months of CAPOX before reassessment, switching to FOLFIRI if still positive and de-escalating to capecitabine if converted; conversion rates after this cross-over were not reported [41].

Two ongoing randomized trials extend the de-escalation question beyond stage II: VEGA (CIRCULATE-Japan) tests whether surgery alone is non-inferior to 3 months of CAPOX in ctDNA-negative high-risk stage II or low-risk stage III colon cancer [164], and CIRCULATE-US (NRG-GI008) randomizes ctDNA-negative patients to immediate chemotherapy versus serial surveillance with delayed treatment [165]. An important caveat to this de-escalation paradigm is provided by the ctDNA-negative cohort of DYNAMIC-III, in which de-escalation to single-agent fluoropyrimidine (versus standard oxaliplatin-based doublet) narrowly missed the prespecified non-inferiority margin for 3-year RFS, although adjuvant chemotherapy-related toxicity was reduced [38]. Taken together, these de-escalation trials are converging on a consistent message—ctDNA negativity defines a low-risk population in whom conventional adjuvant chemotherapy may be safely reduced or omitted—but the precise magnitude of acceptable RFS difference and the optimal substage thresholds remain unsettled in stage III disease and await definitive resolution by phase III readouts from VEGA and CIRCULATE-US.

In contrast to the relative success of ctDNA-guided de-escalation, intensifying therapy in ctDNA-positive patients has proven considerably more challenging. Escalation arises in two distinct contexts that pose different biological questions and have been addressed by distinct trial designs: immediate intensification at the postoperative MRD window (weeks 4–7), and treatment of ctDNA persisting after completion of standard adjuvant chemotherapy.

*Early postoperative ctDNA positivity (immediate adjuvant intensification).* The question here is whether the conventional cytotoxic intensification used in routine practice can convert ctDNA-positive patients into long-term disease-free survivors. DYNAMIC-III tested this in stage III colon cancer in a multicenter phase II/III trial with ctDNA testing 5–6 weeks after surgery [38]. ctDNA-negative patients had markedly better 3-year RFS than ctDNA-positive patients, validating ctDNA as a powerful prognostic stratifier, but escalation in the ctDNA-positive cohort—from single-agent fluoropyrimidine to oxaliplatin-based doublet, and to FOLFOXIRI in selected patients—did not improve RFS over standard care [38,163]. Higher ctDNA molecular burden progressively stratified recurrence risk, and persistent ctDNA after treatment conferred profoundly worse outcomes than ctDNA clearance [38]. Conventional chemotherapy escalation therefore does not overcome ctDNA-positive disease when applied uniformly at the immediate postoperative MRD window, indicating a need for novel strategies [38].

The inadequacy of conventional chemotherapy in this early postoperative context is further illustrated by serial ctDNA kinetics. A prospective multicenter cohort of stage III CRC patients identified two biologically distinct patterns of ctDNA increase—slow-growth and fast-growth—with the fast-growth phenotype independently prognostic of OS; among ctDNA-positive patients receiving adjuvant chemotherapy, recurrence occurred in the majority of those treated with standard regimens regardless of ctDNA kinetics [173]. These findings suggest that ctDNA burden and growth dynamics may identify patients with aggressive residual disease for whom novel rather than intensified conventional strategies are required, and the distinct biology of these two phenotypes may inform future enrichment and biology-directed treatment-matching strategies [173]. The escalation cohort of CIRCULATE-US (NRG-GI008), randomizing ctDNA-positive patients to FOLFOX/CAPEOX versus FOLFIRINOX, remains the principal ongoing test of conventional cytotoxic intensification in this window [165]. Building on the recognition that ctDNA-positive patients respond heterogeneously to identical regimens, the phase II REVISE trial (NCT06242418) introduces a response-adapted paradigm: after 2 cycles of XELOX to establish ctDNA dynamics, ctDNA-persistent patients are randomized to FOLFOXIRI versus continued XELOX, while those clearing ctDNA continue XELOX alone [174]. Its small planned sample size, however, limits statistical power for survival endpoints.

*Persistent ctDNA positivity after completion of standard adjuvant chemotherapy*. The second context—patients who remain ctDNA-positive after completing standard adjuvant chemotherapy—represents a fundamentally different biological setting: residual molecular disease has, by definition, demonstrated resistance to a full course of conventional cytotoxic therapy, motivating evaluation of agents with distinct mechanisms of action and non-cross-resistant pharmacology. Proof-of-concept that trifluridine/tipiracil (FTD/TPI) can modulate ctDNA in this setting came from INTERCEPT-TT, a single-arm phase II trial in patients with ctDNA-defined MRD after curative-intent therapy and adjuvant chemotherapy, compared with a matched synthetic control [40]. The trial met its primary endpoint, with higher 6-month ctDNA clearance and longer median DFS than the synthetic control [40]; the single-arm design, small sample size, synthetic control, and predominance of stage IV disease nonetheless limit generalizability to the perioperative stage II–III setting and motivated phase III validation.

ALTAIR (CIRCULATE-Japan) was the first phase III trial designed specifically for the post-adjuvant ctDNA-persistent context, enrolling 243 ctDNA-positive patients with resected stage II–IV CRC who had completed standard adjuvant chemotherapy but remained ctDNA-positive (confirmed by Signatera, without radiological recurrence), randomized to FTD/TPI versus placebo for 6 months [37]. In the pre-specified analysis, FTD/TPI produced numerically higher median DFS that did not reach statistical significance overall, whereas a post hoc analysis incorporating blinded central radiographic review yielded a nominally significant DFS benefit; an exploratory, multiplicity-unadjusted subgroup analysis suggested a larger effect in resected oligometastatic stage IV disease (HR 0.53) [37]. Together with INTERCEPT-TT, these findings provide the first signal that a novel therapeutic strategy can improve outcomes in patients with persistent ctDNA after completion of standard adjuvant therapy, with the largest apparent effect confined to an exploratory stage IV subgroup requiring confirmation, while confirming that conventional chemotherapy intensification is insufficient in this molecularly resistant population.

Beyond binary ctDNA-positive/negative dichotomization, integrating ctDNA with conventional biomarkers may further refine the population most likely to benefit from escalation in either context. A post hoc analysis of the IDEA-France phase III trial examined whether integrating ctDNA with carcinoembryonic antigen (CEA) and pathological TN (pTN) staging improves risk stratification in stage III colon cancer [175]. A novel three-variable risk classification combining ctDNA, CEA, and pTN reclassified a subset of pT4/N2 patients as lower risk and a smaller subset of pT3/N1 patients as higher risk, with markedly divergent 3-year DFS between the resulting low- and high-risk groups [175]. These data suggest that ctDNA can refine risk stratification beyond conventional staging to inform adjuvant treatment intensity decisions, though prospective validation is required.

Several additional international platforms span both contexts. In Europe, CIRCULATE/AIO-KRK-0217 (NCT04089631) is evaluating ctDNA-guided adjuvant decisions in stage II colon cancer; interim screening showed consistently low postoperative ctDNA positivity (4–6%), underscoring the limited statistical power inherent to ctDNA-guided trials in this substage, with efficacy results pending [176,177]. The Danish IMPROVE-IT2 trial (NCT04084249) evaluates ctDNA-guided surveillance versus standard CT-based follow-up in high-risk stage II–III CRC [178], and in the United Kingdom TRACC Part C is investigating de-escalation using a tissue-free platform, building on the TRACC Part B prognostic dataset [158]. Results from these international platforms, expected in the latter half of the decade, will be essential to determine whether survival benefit from ctDNA-guided escalation can be confirmed across populations and novel therapeutic approaches. Collectively, the escalation trial landscape illustrates an evolving conceptual progression—from failed conventional cytotoxic intensification at the early postoperative MRD window, through early proof-of-concept signals with non-cross-resistant agents in the post-adjuvant persistent setting, toward emerging dynamic kinetics-guided and multi-variable risk-adapted approaches—reflecting the central recognition that ctDNA-positive disease is biologically heterogeneous and unlikely to be overcome by uniform escalation regimens.

### 6.4. ctDNA During and After Neoadjuvant Therapy

The integration of ctDNA monitoring into the neoadjuvant treatment period opens distinct opportunities: predicting response to therapy, guiding organ preservation decisions, and identifying residual disease after treatment completion [179]. In patients with locally advanced MSI-H/dMMR solid tumors—including colorectal cancer—treated with neoadjuvant pembrolizumab, ctDNA clearance after treatment was associated with markedly higher rates of pathological complete response in the colorectal cancer subgroup and with superior event-free survival across all evaluable patients [92]. Prospective data supporting ctDNA-guided neoadjuvant therapy decision-making in MSS/pMMR colon cancer are currently lacking. These questions are particularly relevant to the TNT and W&W paradigms discussed above. Across LARC studies, the most consistent finding is that ctDNA detects MRD after neoadjuvant chemoradiotherapy (nCRT) and surgery, with positivity conferring significantly worse prognosis, whereas its ability to predict pCR during nCRT has been more variable [179].

Serial ctDNA analysis during nCRT for LARC has demonstrated that ctDNA levels decline substantially with effective treatment. In a prospective Chinese multicenter study, ctDNA was detectable in most patients at baseline and progressively declined during nCRT and after surgery, with none of the pCR patients showing detectable ctDNA pre-surgically—establishing the association between ctDNA dynamics during nCRT and pCR [180]. A separate analysis from the UNION phase III trial demonstrated that ctDNA negativity and ctDNA clearance after short-course radiotherapy correlated significantly with pCR, and a predictive model combining ctDNA with CEA outperformed single-marker approaches in predicting pCR status [181]. These observations suggest that ctDNA kinetics during nCRT carry information about tumor sensitivity, though the predictive value for pCR has been variable across studies and is influenced by assay choice and collection timing [160,179].

The NOMINATE trial, a prospective multicenter randomized phase II study of TNT and NOM in locally advanced rectal cancer, incorporated a tumor-informed (Signatera) ctDNA companion analysis with serial sampling from baseline through final re-staging and after surgery or NOM [29]. Baseline detection was near-universal and declined progressively during TNT, with a partial rebound at final re-staging reflecting residual disease in non-responders. Among patients achieving cCR or near-cCR who elected NOM, ctDNA clearance was complete at end-of-TNT and beyond, whereas non-cCR patients showed substantially lower clearance. ctDNA at final re-staging demonstrated very high specificity and positive predictive value for pathologic residual disease and was associated with significantly shorter DFS, but its sensitivity for detecting subsequent local regrowth was modest [29]. This finding is clinically important: while ctDNA positivity after TNT reliably predicts residual disease (and strongly indicates surgical resection), ctDNA negativity does not guarantee absence of local regrowth, highlighting the continued indispensability of endoscopic and radiological surveillance in W&W programs.

In the dMMR rectal cancer setting, neoadjuvant immunotherapy with PD-1 blockade yields unprecedented clinical complete response rates approaching 100% in selected series [22,31]. Although dMMR tumors demonstrate lower ctDNA shedding in the postoperative minimal residual disease setting (as described above), bulky locally advanced dMMR rectal cancers show near-universal baseline ctDNA detectability in both the NOMINATE cohort [29] and the dostarlimab phase II study [22], making ctDNA clearance during treatment a meaningful and trackable biomarker. ctDNA clearance in this context occurs rapidly and shows high concordance with biopsy-confirmed response [22]. Among colorectal cancer patients managed non-operatively, ctDNA-undetectable status after immunotherapy was associated with markedly superior event-free survival compared with persistent ctDNA positivity [92]. These data suggest that ctDNA clearance kinetics during immunotherapy may provide early confirmation of deep response and reinforce the decision for organ preservation, though an educational review confirmed that ctDNA cannot yet replace definitive pathologic assessment outside of clinical trials [162]. Prospective validation of ctDNA-guided NOM after immunotherapy in dMMR rectal cancer is an active area of investigation.

A ctDNA-guided neoadjuvant strategy for LARC is being prospectively evaluated in the CINTS-R trial (NCT05601505), a multicenter randomized controlled trial [167]. Against a standard nCRT control arm, the experimental arm stratifies treatment by ctDNA status and molecular profile: ctDNA-defined high-risk MSS/pMMR patients receive intensified TNT, ctDNA-defined low-risk patients proceed directly to surgery after nCRT, and MSI-H/dMMR or high-TMB tumors receive neoadjuvant tislelizumab regardless of ctDNA. An interim analysis confirmed acceptable safety, although a notable proportion of TNT-treated patients discontinued chemotherapy for adverse events [168]. CINTS-R is the first prospective randomized attempt to personalize the intensity and modality of neoadjuvant therapy in LARC by ctDNA; efficacy outcomes are awaited.

A complementary observation comes from the phase I/II TARC trial, in which neoadjuvant trifluridine/tipiracil-based chemoradiotherapy in LARC achieved pathological downstaging in nearly all evaluable patients (one pCR; one cCR proceeding to W&W); however, ctDNA detected residual viable tumor in only a subset of evaluable patients before surgery [182]. Taken together, these neoadjuvant studies indicate that ctDNA provides robust prognostic information after nCRT and during immunotherapy-driven organ preservation, but its sensitivity for detecting microscopic residual disease and local regrowth in the W&W context remains insufficient to replace endoscopic and radiological surveillance.

Beyond response assessment during definitive nCRT, ctDNA-guided postoperative surveillance has emerged as a distinct application. The phase III FIND trial randomized 728 patients with non-metastatic resected CRC to ctDNA methylation-based surveillance versus standard imaging-based follow-up, with the proportion of recurrent patients receiving curative-intent therapy within 2 years as the primary endpoint [39]. In the interim analysis, cumulative recurrence rates did not differ between the arms, whereas the proportion of recurrent patients subsequently undergoing curative-intent treatment was significantly higher in the ctDNA-guided arm [39]. These interim data represent the first randomized phase III signal that ctDNA-based surveillance translates into a higher rate of curative-intent salvage treatment; whether this ultimately improves survival remains to be demonstrated and mature results are awaited, but the finding supports continued prospective evaluation of ctDNA monitoring as an adjunct to conventional imaging.

### 6.5. ctDNA Monitoring After Curative-Intent Resection of Colorectal Liver Metastases

The ctDNA MRD framework has been extended to curative-intent resection of colorectal liver metastases (CLMs), where adjuvant chemotherapy decisions remain controversial and which fall within the curative-intent perioperative continuum rather than the palliative setting. In a CIRCULATE-Japan GALAXY subgroup undergoing upfront hepatectomy without preoperative chemotherapy or extrahepatic disease, postoperative ctDNA positivity at week 4 was 32.1% (61/190)—roughly double that in primary resectable CRC [36]—consistent with the higher disease burden [183]. MRD positivity conferred markedly inferior DFS and retained independent prognostic value on multivariate analysis, and the benefit of adjuvant chemotherapy was strikingly differential, being confined to MRD-positive patients [183]. An updated analysis with longer follow-up extended these observations to overall survival, with MRD-positive patients deriving both disease-free and overall survival benefit from adjuvant chemotherapy, whereas MRD-negative patients had favorable survival without clear benefit [184]. These observations suggest that postoperative ctDNA status may identify the subset of CLM patients who derive meaningful benefit from adjuvant chemotherapy while sparing MRD-negative patients from unnecessary cytotoxic exposure. Whether ctDNA-guided adjuvant decisions after CLM resection can reduce unnecessary systemic chemotherapy in this high-risk but heterogeneous population remains to be determined in dedicated RCT evidence, and represents a logical next frontier for the ctDNA-guided perioperative framework established in non-metastatic disease.

### 6.6. Challenges for Clinical Integration and Future Directions

A consistent theme emerging from the prognostic and interventional evidence reviewed above is that ctDNA results should not be interpreted in isolation when adjuvant management decisions are being made. Clinicopathological factors—including CEA, pathological TN stage, histological grade, mismatch repair status, and primary tumor sidedness—retain independent prognostic value, and composite biomarker frameworks have been shown to refine risk stratification beyond ctDNA alone, as illustrated by the IDEA-France post hoc three-variable model combining ctDNA, CEA, and pTN [175] and the HIBRID model integrating ctDNA with deep learning-based histological risk assessment [185]. Accordingly, ctDNA is best regarded as one component of a multi-variable risk assessment rather than a standalone determinant of adjuvant therapy.

Despite the rapid maturation of the prognostic evidence base, several practical and conceptual barriers continue to limit the routine integration of ctDNA testing into perioperative CRC management outside of clinical trials. Clinical integration faces several practical barriers, including limited assay interoperability across platforms (Signatera, RaDaR, Guardant Reveal, and methylation-based assays differ in technology, panels, and analytical cutoffs), inconsistent reimbursement across health systems, and the absence of standardized protocols for collection timing and result reporting [157,186]. Clinician education regarding the distinction between prognostic information and actionable therapeutic guidance remains essential before widespread implementation.

Current ctDNA assays rely primarily on somatic mutation detection using whole-exome or targeted sequencing, but next-generation WGS-based tumor-informed assays offer the potential to achieve detection sensitivity at the parts-per-million level, substantially improving capture of low-shedding tumors such as those with dMMR or pulmonary recurrence patterns. The SCRUM-Japan MONSTAR-SCREEN-3 platform (UMIN000053975), which incorporates a WGS-based MRD assay into a dedicated cohort of resectable solid tumors, including CRC, represents the first systematic evaluation of this approach in the perioperative setting [161]. Simultaneously, emerging multi-analyte approaches—incorporating methylation patterns, cfDNA fragmentomics, and nucleosome modification profiles alongside mutation signals—may further improve sensitivity and specificity [187]. A complementary advance involves integrating ctDNA results with other data modalities. The HIBRID study combined ctDNA MRD status from GALAXY with deep learning analysis of H&E whole-slide images, demonstrating that the combined approach improved prognostic stratification beyond either modality alone: ctDNA-negative patients with high-risk histological features identified by the deep learning model derived significant adjuvant chemotherapy benefit that ctDNA status alone did not predict [185]. Together, these multi-signal approaches may ultimately enable more precise individualization of adjuvant therapy decisions and surveillance intensity than any single biomarker.

Despite substantial progress, several foundational challenges remain before ctDNA testing can be considered a mature clinical tool universally integrated into perioperative CRC management. First, the sensitivity of ctDNA assays for low-volume MRD remains imperfect, with false negative rates that are especially problematic in dMMR, right-sided, and early-stage tumors [35,162]. Second, the demonstration that ctDNA-guided escalation improves survival outcomes remains incomplete: while ALTAIR provided an early signal of benefit with trifluridine/tipiracil in ctDNA-positive patients, the prespecified primary analysis did not reach statistical significance and results of CIRCULATE-US cohort B remain awaited [37,38,165]. Third, the cost of tumor-informed assays, together with the multiple serial collections required, raises questions about cost-effectiveness and equitable access in resource-limited settings [157]. Fourth, practical implementation—including assay turnaround time, laboratory accreditation, clinician interpretation, and patient communication—has not been systematically studied in most healthcare systems [160]. Fifth, in resected stage II colon cancer, postoperative ctDNA was detected in only 7.9% (14/178) of patients not treated with adjuvant chemotherapy in the foundational cohort [169], limiting the statistical power of trials in this substage.

The field is therefore at an inflection point. The prognostic value of postoperative ctDNA is now well established across multiple prospective cohorts, trial substudies, and meta-analyses [35,36,160,166,169]. The therapeutic utility—whether ctDNA-guided treatment decisions translate into improved survival—is the frontier that ongoing phase III trials must address. Collaborative efforts toward assay standardization, health economic modeling, and trial designs that integrate ctDNA with both conventional and novel therapeutic agents will be essential to realize the full potential of ctDNA-based MRD assessment in the perioperative management of colorectal cancer [188].

Pivotal ctDNA-guided clinical trials in perioperative colorectal cancer are summarized in Table 6.

**Table 6:** Pivotal Trials of ctDNA-Guided Perioperative Therapy in Colorectal Cancer.

Trial (Year)	N	Setting/Stage	Strategy	Assay (TI/TA)	Primary Endpoint	Key Result	Reference	Level of Evidence ‡
DYNAMIC (2022; Randomized Ph II)	455	II colon	De-escalation	TI (Safe-SeqS)	2 yr RFS (non-inferiority)	2 yr RFS 93.5% vs. 92.4%; difference 1.1 pp (95% CI −4.1 to 6.2); adj chemo use 15% vs. 28%	[33]	Guideline-supported emerging (de-escalation)
— DYNAMIC 5 yr (2025; Randomized Ph II)	455	II colon	De-escalation	TI	5 yr RFS and OS	5 yr RFS 88.3% vs. 87.2%; 5 yr OS 93.8% vs. 93.3%; HR 1.05, 95% CI 0.47–2.37; *p* = 0.887	[34]	Guideline-supported emerging (de-escalation)
IDEA-France post hoc (2024; post-hoc)	—	III colon	Risk stratification (ctDNA + CEA + pTN)	TA (methylation ddPCR)	3 yr DFS by risk class	3 yr DFS low-risk 80.8% vs. high-risk 55.4%; HR 2.66, 95% CI 1.84–3.86; *p* < 0.001	[175]	Supportive (post hoc)
GALAXY (CIRCULATE-Japan, 2024; observational)	2240	II–IV CRC	Prognostic	TI (Signatera)	DFS, OS by ctDNA status	ctDNA+ DFS HR 11.99, 95% CI 10.02–14.35; *p* < 0.0001; adj chemo in ctDNA+ only, adjusted HR 0.23, 95% CI 0.15–0.35	[35,36]	Prognostic: established
— GALAXY CLM subgroup (2024; observational subgroup)	190	Resected colorectal liver metastases	Prognostic + adj chemo response	TI (Signatera)	DFS by ctDNA status	24-mo DFS 10.8% (MRD+) vs. 64.5% (MRD−); adj chemo in MRD+ only, adjusted HR 0.07, 95% CI 0.02–0.26; *p* < 0.0001	[183]	Prognostic: established
— GALAXY CLM update (2026; observational subgroup)	298	Resected colorectal liver metastases	Prognostic + adj chemo response	TI (Signatera)	DFS, OS by ctDNA status	Median follow-up 43.2 mo; adjuvant chemotherapy benefit confined to MRD+ patients, extending to OS: OS HR 0.27, 95% CI 0.08–0.88; *p* = 0.03	[184]	Prognostic: established
— GALAXY rectal subgroup (2026; observational subgroup)	246 evaluable	II–III rectal upfront surgery	Prognostic + adj chemo response	TI (Signatera)	DFS by ctDNA status	DFS HR 9.96, 95% CI 5.76–17.2; *p* < 0.0001; adj chemo in ctDNA+ only, HR 0.28, 95% CI 0.09–0.89; *p* = 0.031	[170]	Prognostic: established
DYNAMIC-III (2025; Ph II/III)	968 evaluable	III colon	Escalation (ctDNA+); de-escalation (ctDNA–)	TI	2 yr RFS (ctDNA+ cohort)	2 yr RFS escalation vs. SOC 51% vs. 61%; HR 1.16, 95% CI 0.87–1.53; 3 yr RFS FOLFOXIRI vs. FOLFOX/CAPOX 47% vs. 51%; HR 1.09, 90% CI 0.78–1.53; *p* = 0.7; ctDNA– de-escalation 3 yr RFS 85.3% vs. 88.1% (NI not met)	[38,163]	Investigational (escalation, negative)
PEGASUS (ESMO 2025, Abstract 723O; Ph II)	135	II high-risk + III colon	De-escalation	TA (Guardant Reveal vL1.2)	2 yr NED in ctDNA– (feasibility threshold <14 relapses among 134 LB− patients)	Median follow-up 33.5 mo; 12 relapses/100 LB-; 2 yr NED 88% (90% CI 81–93); 2 yr time-to-relapse 87.6% (LB-) vs. 61.5% (LB+); HR 3.22, 95% CI 1.20–7.96; *p* = 0.0013	[41]	Investigational (de-escalation)
ALTAIR (2026; Ph III)	243	II–IV ctDNA+ CRC	Escalation post adjuvant	TI (Signatera)	DFS	mDFS 9.30 vs. 5.55 mo; HR 0.79, 95% CI 0.60–1.05; *p* = 0.107 (overall); post hoc HR 0.75, 95% CI 0.55–0.98; *p* = 0.041 (blinded central review)	[37]	Investigational (escalation signal)
INTERCEPT-TT (2025; Ph II)	15 (synthetic ctrl 30; 2:1)	II–IV CRC, ctDNA+ MRD post-adjuvant	Escalation (TAS-102)	TI (Signatera)	ctDNA clearance at 6 mo	ctDNA clearance 33% vs. 6.7%; *p* = 0.025; mDFS 9.41 vs. 5.75 mo; HR 0.47, 95% CI 0.23–0.85; *p* = 0.03	[40]	Investigational (single-arm)
NOMINATE ctDNA analysis (2025; Randomized Ph II)	64 evaluable	Distal LARC	Neoadjuvant + NOM monitoring	TI (Signatera)	ctDNA dynamics, DFS	ctDNA at final re-staging: 100% specificity for residual disease; DFS HR 6.70, 95% CI 1.5–29.7; *p* = 0.005	[29]	Investigational (prognostic)
FIND (Peng 2025, ESMO GI; Ph III, interim)	728	Non-metastatic resected CRC	Surveillance	TA (methylation)	Proportion of recurrent patients receiving curative-intent therapy within 2 yr	Curative-intent treatment 50.0% (95% CI 31.4–68.6) vs. 22.6% (95% CI 11.4–39.8); RR 2.21, 95% CI 1.06–4.78; *p* = 0.034; no difference in cumulative recurrence rate	[39]	Investigational (phase III interim)
**— Ongoing trials —**								
VEGA (jRCT1031200006; Ph III)	TBD	II high-risk/III low-risk colon	De-escalation (CIRCULATE-Japan)	TI (Signatera)	DFS (non-inferiority of surgery alone vs. CAPOX × 3 in ctDNA–)	TBD	[164]	Ongoing
CIRCULATE-US (NRG-GI008, NCT05174169; Ph II/III)	TBD	II–III colon	De-escalation + escalation	TI (Signatera)	DFS	Pairs concurrent de-escalation and escalation cohorts	[165]	Ongoing
CIRCULATE/AIO-KRK-0217 (NCT04089631; Ph III)	1439 screened	II colon	De-escalation	TI	DFS	Recruitment ongoing across >140 centers in Germany and Austria; ctDNA+ rate 4–6% in screening data	[176,177]	Ongoing
CINTS-R (NCT05601505; Randomized Ph II)	316 (interim)	LARC	Neoadjuvant; ctDNA-guided intensification	TI	Final efficacy endpoints pending	Interim safety reported: serious AE 10.0% vs. 6.6%; *p* = 0.316; 15.7% TNT discontinuation	[167,168]	Ongoing
REVISE (NCT06242418; Ph II)	60 (planned)	III colon, ctDNA+ within 1 mo post-surgery	Dynamic escalation: intensified FOLFOXIRI vs. standard XELOX in ctDNA-persistent patients	TI	ΔctDNA (primary); DFS (secondary)	TBD	[174]	Ongoing

*Note:* Ongoing trials are listed with NCT registry identifiers. TBD = to be determined. *Abbreviations:* adj, adjuvant; AE, adverse event; CAPOX, capecitabine plus oxaliplatin; CEA, carcinoembryonic antigen; CI, confidence interval; CLM, colorectal liver metastasis; CRC, colorectal cancer; ctDNA, circulating tumor DNA; DFS, disease-free survival; FOLFOXIRI, fluorouracil/leucovorin/oxaliplatin/irinotecan; HR, hazard ratio; LARC, locally advanced rectal cancer; LB, liquid biopsy; mDFS, median disease-free survival; MRD, minimal residual disease; NED, non-evidence of disease; NI, non-inferiority; NOM, non-operative management; OS, overall survival; pp, percentage point; pTN, pathological tumor-node stage; RFS, recurrence-free survival; RR, rate ratio; SOC, standard of care; TA, tumor-agnostic; TAS-102, trifluridine/tipiracil; TBD, to be determined; TI, tumor-informed; TNT, total neoadjuvant therapy; XELOX, capecitabine plus oxaliplatin. ‡ Evidence-level classification as defined in the footnote to Table 1.

## 7. Conclusions

Perioperative strategies for CRC are moving from uniform treatment toward biology-, site-, and risk-adapted care. The strength of the underlying evidence, however, varies markedly across strategies, and each is best regarded as an established standard, a guideline-supported emerging option, an investigational approach, or an ongoing trial; distinguishing these levels is essential when translating these advances into practice. Ongoing randomized trials will be decisive in determining which emerging strategies become future standards. The next phase should focus on precise patient selection, the development of novel therapeutic strategies, and the preservation of function. ctDNA is a robust prognostic marker, but its clinical utility for guiding therapy—particularly treatment escalation—requires prospective validation before routine, universal implementation.

## Figures and Tables

**Table 1:** Perioperative Treatment Framework by Mismatch Repair Status and Primary Site, with ctDNA-MRD as a Cross-Cutting Concept.

Category	Site	MMR Status	Approx. Prevalence †	Perioperative Treatment Paradigm	Key Pivotal Trials	Section	Level of Evidence ‡
MSS/pMMR colon	Colon	MSS/pMMR	~85–90% of non-metastatic colon cancer	Oxaliplatin-based adjuvant chemotherapy; risk-adapted duration; neoadjuvant chemotherapy emerging	MOSAIC [2,12]; NO16968/XELOXA [3]; IDEA [4]; FOxTROT [13,14]	Section 2	Established standard (adjuvant chemotherapy, risk-adapted duration); guideline-supported emerging (neoadjuvant chemotherapy in cT4b or bulky nodal pMMR disease [24])
MSI-H/dMMR colon	Colon	MSI-H/dMMR	~10–15% of non-metastatic colon cancer	Adjuvant ICI plus chemotherapy; short-course neoadjuvant dual ICI	NICHE-1 [9]; NICHE-2 [10,15]; NICHE-3 [16]; ATOMIC [17]; RESET-C [18]	Section 3	Guideline-supported emerging (adjuvant ICI); investigational (neoadjuvant ICI, non-operative management)
MSS/pMMR rectal	Rectum	MSS/pMMR	~90–95% of rectal cancer	Total neoadjuvant therapy (TNT); watch-and-wait organ preservation; selective RT omission; emerging immune-radiation strategies	CAO/ARO/AIO-12 [25]; PRODIGE 23 [6,20]; RAPIDO [5,21]; OPRA [7,26]; PROSPECT [8]; UNION [27]; NOMINATE [28,29]; STELLAR II [30]	Section 4	Established standard (TNT, selective RT omission in MRF-negative disease); guideline-supported emerging (watch-and-wait); investigational (immune-radiation strategies)
MSI-H/dMMR rectal	Rectum	MSI-H/dMMR	~5–10% of rectal cancer	Neoadjuvant immunotherapy-based strategies for organ preservation	Cercek dostarlimab [31]; Cercek dostarlimab update [22]; RESET-R [32]	Section 5	Guideline-supported emerging (immunotherapy, watch-and-wait); evidence based on single-arm data
**ctDNA-MRD assessment** *(cross-cutting)*	All sites	All MMR	—	Adaptive treatment escalation/de-escalation; surveillance; response monitoring during neoadjuvant treatment	DYNAMIC [33,34]; GALAXY [35,36]; ALTAIR [37]; DYNAMIC-III [38]; FIND [39]; INTERCEPT-TT [40]; PEGASUS [41]	Section 6	Prognostic value established; de-escalation supported by randomized data in stage II (thresholds unsettled in stage III); therapeutic escalation investigational

† Approximate prevalence in the resectable (stage II–III) setting; colon dMMR prevalence ~15–18% in stage II and ~9–10% in stage III [42]; rectal dMMR prevalence ~5–10% [31]. The frequency of MSI-H/dMMR tumors is stage-dependent and decreases substantially in metastatic disease (~4–5% in stage IV colorectal cancer) [42]. ‡ Evidence-level classification. Four tiers are applied to treatment strategies (*Established standard*; *Guideline-supported emerging*; *Investigational*; *Ongoing*); two additional descriptors (*Historical*, *Supportive*) are applied to individual trials in the subsequent tables. Definitions (based on trial phase, guideline incorporation, and maturity of survival/durability data): *Established standard* = supported by phase III evidence and incorporated into guidelines as standard of care; *Guideline-supported emerging* = incorporated into guidelines but with immature overall survival or long-term durability data; *Investigational* = supported mainly by single-arm phase II or early-phase data in selected populations; *Ongoing* = under evaluation in active randomized trials; *Historical* = foundational or superseded trials of primarily historical importance; *Supportive* = confirmatory or supportive analyses (e.g., pooled or real-world data) that inform but do not by themselves define practice. *Abbreviations:* ctDNA, circulating tumor DNA; dMMR, deficient mismatch repair; ICI, immune checkpoint inhibitor; MMR, mismatch repair; MRD, minimal residual disease; MSI-H, microsatellite instability-high; MSS, microsatellite stable; pMMR, proficient mismatch repair; RT, radiotherapy; TNT, total neoadjuvant therapy.

## Data Availability

No new data were created or analyzed in this study. Data sharing is not applicable to this article.
